# CHK2 activation contributes to the development of oxaliplatin resistance in colorectal cancer

**DOI:** 10.1038/s41416-022-01946-9

**Published:** 2022-08-23

**Authors:** Chi-Che Hsieh, Sen-Huei Hsu, Chih-Yu Lin, Hung-Jiun Liaw, Ting-Wei Li, Kuan-Ying Jiang, Nai-Jung Chiang, Shang-Hung Chen, Bo-Wen Lin, Po-Chuan Chen, Ren-Hao Chan, Peng-Chan Lin, Yu-Min Yeh, Che-Hung Shen

**Affiliations:** 1 National Institute of Cancer Research, National Health Research Institutes, Tainan, 704 Taiwan; 2 Department of Life Sciences, National Cheng Kung University, Tainan, 701 Taiwan; 3 Department of Oncology, Taipei Veterans General Hospital, Taipei, 112 Taiwan; 4 School of Medicine, National Yang Ming Chiao Tung University, Taipei, 112 Taiwan; 5 Department of Oncology, National Cheng Kung University Hospital, College of Medicine, National Cheng Kung University, Tainan, 704 Taiwan; 6 Department of Surgery, National Cheng Kung University Hospital, College of Medicine, National Cheng Kung University, Tainan, 704 Taiwan; 7 Department of Genomic Medicine, National Cheng Kung University Hospital, College of Medicine, National Cheng Kung University, Tainan, 704 Taiwan; 8 Department of Computer Science and Information Engineering, College of Electrical Engineering and Computer Science, National Cheng Kung University, Tainan, 701 Taiwan; 9 Ph.D. Program in Tissue Engineering and Regenerative Medicine, Biotechnology Center, National Chung Hsing University, Taichung, 402 Taiwan

**Keywords:** Cancer therapeutic resistance, Checkpoint signalling

## Abstract

**Background:**

Colorectal cancer (CRC), the most common cancer type, causes high morbidity and mortality. Patients who develop drug resistance to oxaliplatin-based regimens have short overall survival. Thus, identifying molecules involved in the development of oxaliplatin resistance is critical for designing therapeutic strategies.

**Methods:**

A proteomic screen was performed to reveal altered protein kinase phosphorylation in oxaliplatin-resistant (OR) CRC tumour spheroids. The function of CHK2 was characterised using several biochemical techniques and evident using in vitro cell and in vivo tumour models.

**Results:**

We revealed that the level of phospho-CHK2(Thr68) was elevated in OR CRC cells and in ~30% of tumour samples from patients with OR CRC. We demonstrated that oxaliplatin activated several phosphatidylinositol 3-kinase-related kinases (PIKKs) and CHK2 downstream effectors and enhanced CHK2/PARP1 interaction to facilitate DNA repair. A phosphorylation mimicking CHK2 mutant, CHK2T68D, but not a kinase-dead CHK2 mutant, CHK2D347A, promoted DNA repair, the CHK2/PARP1 interaction, and cell growth in the presence of oxaliplatin. Finally, we showed that a CHK2 inhibitor, BML-277, reduced protein poly(ADP-ribosyl)ation (PARylation), FANCD2 monoubiquitination, homologous recombination and OR CRC cell growth in vitro and in vivo.

**Conclusion:**

Our findings suggest that CHK2 activity is critical for modulating oxaliplatin response and that CHK2 is a potential therapeutic target for OR CRC.

## Introduction

Colorectal cancer (CRC) is a disease arising from the malignant transformation of a benign polyp. The accumulation of genetic mutations and epigenetic alterations promote tumour cell proliferation and distant metastases in the advanced stage of CRC development [1]. CRC is the third leading cause of cancer-related deaths worldwide. It is expected to cause ~52,580 deaths during 2022 [2]. Despite extensive efforts in public health policies and improvement of clinical diagnosis and treatment of CRC, the significantly increasing number of new cases and the high mortality of this cancer remain major concerns.

The curative treatment of CRCs can be achieved by surgery, radiation, or chemotherapy. Chemotherapy is used as adjuvant therapy after surgery or as neoadjuvant therapy to shrink a tumour before removal. In the past decade, various chemotherapeutic combinations have significantly improved the median survival of patients with recurrent and advanced CRC [3]. Oxaliplatin is a third-generation platinum compound that mainly interacts with DNA to induce the formation of Pt-DNA adducts (intra- and inter-strand crosslinks) [4]. These adducts strongly block DNA replication, leading to replication fork collapse and the formation of lethal DSB that account for the therapeutic efficacy of oxaliplatin. It also revealed that oxaliplatin interferes with RNA synthesis in mouse leukaemia cells and modifies proteins, resulting in loss of function, dysregulation of the protein network, and cell apoptosis [5, 6]. Oxaliplatin is recommended as a first-line therapeutic regimen for metastatic colorectal cancer. It is usually combined with other drugs, such as folinic acid, 5-FU + oxaliplatin (FOLFOX), and folinic acid + 5-FU + irinotecan (FOLFIRI) [7–9]. However, CRC patients responding to these oxaliplatin-based regimens develop drug resistance with severe adverse events and have a median overall survival of up to 24 months [7–10]. Thus, new therapeutic strategies are needed.

Inter-strand crosslinks (ICLs)-induced DNA lesions are primarily repaired by a Fanconi anaemia (FA) pathway in concert with translesion DNA synthesis (TLS) polymerase, nucleotide excision repair (NER), and homologous recombination (HR) repair pathways in mammalian cells [11]. At least 15 genes are involved in the FA pathway. Eight FA proteins (FANCA/B/C/E/F/G/L/M) form a ubiquitin E3 ligase, which monoubiquitinates FANCD2 and FANCI after DNA damage treatment [12]. The monoubiquitinated FANCD2 acts as a platform to recruit DNA nucleases, including MUS81-EME1, XPF-ERCC1 and FAN1, which catalyse ICL unhooking. The unhooking process leaves DNA adducts attached to the complementary strand, which is bypassed by TLS polymerases and the DNA adducts are finally removed by NER. In addition, the unhooking process generates DSBs, which are repaired through HR [11]. Ataxia-telangiectasia mutated (ATM), Rad3-related protein (ATR), and DNA-dependent protein kinase catalytic subunit (DNA-PKcs) belong to the phosphatidylinositol 3-kinase-related kinases (PIKKs) family and coordinate DNA repair process and cell cycle progression to preserve genomic stability against tumorigenesis and tumour progression [13]. ICLs-induced DNA damage blocks DNA replication, leading to replication stress, replication fork collapse, and DSBs. ATR is rapidly activated in response to replication stress, whereas ATM is activated several hours later once DSBs actually appear, resulting from fork collapse [14]. DNA-PKcs is also activated by DSBs, which induce the autophosphorylation of DNA-PKcs at Ser2056 and have been widely used as the activation marker for DNA-PKcs. Moreover, DNA-PKcs phosphorylation at Thr2609 cluster has been suggested to be phosphorylated by ionising radiation-activated ATM kinase or by UV irradiation-activated ATR [15, 16].

Checkpoint kinase 2 (CHK2) is central to transducing the DNA damage signal and functions in maintaining genomic stability. It is well established that CHK2 is phosphorylated and activated by ATM in response to DSBs and activated by ATR in response to replication stress [17, 18]. DNA-PKcs activity has been associated with the phosphorylation and activation of CHK2 in response to DSBs [19, 20], but the interaction of DNA-PKcs and CHK2 in response to oxaliplatin has not yet been revealed. The phosphorylation of CHK2 on certain residues, including Thr68, located at the SCD (SQ/TQ cluster) domain changes the conformation of CHK2, which induces transdimerization of CHK2 and autophosphorylation at Ser516, leading to full CHK2 activation [21, 22]. Studies have revealed that CHK2 phosphorylates downstream effectors, such as p53, cell division cycle 25 (CDC25), and poly (ADP-ribose) polymerase 1 (PARP1), to induce cell cycle arrest, apoptosis and DNA damage repair [17, 23].

Oxaliplatin-induced DSBs cause fatal damage to CRC cells. In this study, we revealed that the phosphorylation levels of CHK2 at Thr68 (pCHK2T68) were elevated in oxaliplatin-resistant (OR) CRC cells and tumour samples. A high level of homologous recombination activity, upregulated phosphorylation levels of CHK2 upstream kinases and downstream effectors, and elevated levels of protein PARylation were observed in OR CRC cells. We found that CHK2T68D, a pCHK2T68 mimic (but not CHK2D347A, a kinase-dead mutant) accelerated oxaliplatin-induced DNA damage repair, enhanced the CHK2/PARP1 interaction, and promoted cell colony formation and xenograft CRC growth in the presence of oxaliplatin. However, these OR CRC cells were resistant to the PARP1 inhibitor, Olaparib. We observed that the FA pathway was upregulated in OR CRC cell lines and was downregulated by knockdown of CHK2 or treatment of the CHK2 inhibitor BML-277. BML-277 was able to inhibit HR and suppress OR CRC cell growth in vitro and in vivo. Our findings suggest that CHK2 activity is critical for CRC cells overcoming oxaliplatin and CHK2 is a potential therapeutic target for OR CRC.

## Materials and methods

### Patient samples and IHC analysis

Patients (*n* = 16) provided signed informed consent prior to their inclusion in this study which was approved by the institutional review board at the National Cheng Kung University Hospital (B-BR-106-068). Tumour biopsies were performed pretreatment and at the time of progressions. Formalin-fixed tissue was analysed to confirm that viable tumour was present via hematoxylin and eosin (H&E) staining. No statistical method was used to predetermine sample size for the IHC analysis. No samples were excluded in the IHC analysis. The investigators were blinded to group allocation and outcome assessment.

For IHC analysis, four-μm-thick sections were prepared and immerse in antigen unmasking buffer pH 9.0 (Vector Laboratories) for antigen retrieval. The sections were subjected with 3% H_2_O_2_ to eliminate endogenous peroxidase activity and blocked with PBS containing 5% of FBS and 0.5% of Triton X-100 before manual staining with primary antibodies: anti-phospho-CHK2 (pThr68) (pCHK2T68, 1:100, C13C1, Cell Signaling Technology), anti-Ki67 (1:200, D2H10, Cell Signaling Technology), and anti-Poly ADP-ribose (1:200, clone 10H, Millipore), followed by incubation with the Dako Real Envision HRP/DAB detection reagent (Dako) according to the manufacturer’s instructions. All sections were counterstained with hematoxylin (Muto Pure Chemicals) and mounted with the Malinol medium (Muto Pure Chemicals). The stained tissues were photographed using a light microscope (the gain was set to 1.0x, saturation to 1.00, and gamma to 1.01, Leica DM2000), and their histopathological characteristics were interpreted by two independent investigators. The expression of pCHK2T68 was semiquantitatively evaluated using the H-score [24] by two independent investigators. The staining intensity was classified into no staining (0), weakly positive [1], moderately positive [2] and strongly positive [3]. The H-score was calculated by multiplying the percentage of stained cells (0.00–1.00) by staining intensity ranging from 0 to 3, resulting in scores from 0 to 3.00.

### Cell culture, transfection and retroviral and lentiviral infection

Human colorectal cancer cell lines: HT29, LoVo, Colo201 and Colo205 cells were provided by Dr. Shang-Hung Chen from ATCC. HT29 cells were kept in McCoy’s 5 A (Sigma); Colo201, Colo205 and LoVo cells were cultured in RPMI1640 (HyClone); 293 cells were maintained in DMEM (HyClone) containing 10% foetal bovine serum (FBS; Hyclone) and 100 U/ml penicillin/streptomycin (Gibco). All cell lines tested negatively for mycoplasma with the MycoSensor PCR Assay Kit (Agilent Technologies).

To generate oxaliplatin-resistant CRC cells, HT29, Colo201, Colo205 and LoVo cell lines were chronically treated with stepwise increased concentration of oxaliplatin. Cells were selected at each step until they formed colonies before moving to the next step. Throughout the 2–3 months of treatment, the oxaliplatin concentration was increased from 0.1 μM to 1.0 μM for oxaliplatin-resistant (OR) group of cells. OR CRC cell lines in this study were maintained in growth medium containing 1 µM oxaliplatin. For tumour spheres generation, individual cells were embedded by culture medium containing 10% Matrigel and grown as tumour spheres before collection for further examinations.

Transfection, retroviral and lentiviral infection were performed as previously described [25]. Briefly, 293 cells were polyethylenimine (PEI) transfected with Ampho packaging vector and pBabe-Puro retroviral vector encoding the gene of interest to produce retroviruses, or with packaging plasmids encoding VSV-G, gag-pol, Rev, and pLKO lentiviral vector encoding shRNA against CHK2 to produce lentiviruses. Culture supernatants containing virus were collected and filtered 48 h post-transfection to infect cultured cells in the presence of 2.5 mg/ml polybrene (Sigma-Aldrich). When indicated, stable populations were obtained and maintained by selection with puromycin (Sigma-Aldrich).

### In vivo xenograft tumour growth studies

Mice were maintained in accordance with facility guidelines on animal welfare and with protocols approved by the Institutional Animal Care and Usage Committee (IACUC) of the National Cheng Kung University. Four- to 6-week-old female NOD/SCID mice (NCKU, Tainan, Taiwan) were housed in a specific pathogen-free environment in the animal facility of NCKU. For oxaliplatin sensitivity assays, 3 × 10^6^ of HT29 cells carrying an empty vector or expressing Flag-tagged CHK2 (F-CHK2) constructs were mixed with Matrigel (1:1, BD Biosciences) and subcutaneously inoculated into the flanks of the mice. When the tumour size reached 100 mm^3^, mice were randomly assigned to two groups: mock and oxaliplatin by the Research Randomizer at http://www.randomizer.org. No statistical method was used to predetermine sample size. The animals were intraperitoneally injected PBS (mock, *n* = 5) or OXA (oxaliplatin 5 mg/kg in PBS, *n* = 5) once per week for 3 weeks. 3 × 10^6^ of Colo205-OR or its oxaliplatin-sensitive counterpart Colo205-P cells were grown as xenograft tumours. The Colo205-OR tumour-bearing mice (*n* = 8) were administrated oxaliplatin (5 mg/kg) once per week for 3 weeks when tumour size reached 100 mm^3^. The Colo205-P tumour-bearing mice were grouped and administrated PBS (mock, *n* = 8) or OXA (oxaliplatin 5 mg/kg, *n* = 8) once per week for 2 weeks when tumour size reached 100 mm^3^.

For BML-277 treatment, the Colo205-OR tumour-bearing mice were randomly assigned to three groups: oxaliplatin 5 mg/kg (*n* = 6), oxaliplatin 5 mg/kg + BML-277 1 mg/kg (*n* = 6) and oxaliplatin 5 mg/kg + BML-277 3 mg/kg (*n* = 6). The animals were intraperitoneally injected oxaliplatin once per week combined with sunflower oil or BML-277. BML-277 was dissolved in DMSO, diluted in sunflower oil, and injected thrice per week for 2 weeks.

The tumours were monitored every day and harvested as indicated. The tumour size was calculated as volume = [length × (width)^2^]/2. The relative tumour volume was calculated as oxaliplatin-treated tumour volume/mock-treated tumour volume. The mice were euthanized when the tumour size reached 1000 mm^3^. The investigators were not blinded to group allocation or outcome assessment. No animals were excluded in these experiments. Mouse tissue sections were analysed as same as patient samples described above.

### Statistics

Statistical analyses were performed using Prism 8 (GraphPad Software). The in vitro experiments were done in biological triplicate each time and independently repeated at least three times. Data are presented as the mean ± SEM and the number (*n*) of samples used was as indicated. Unpaired two-tailed Student’s *t* test was used to compare differences between the control and experimental groups. For all statistical analyses, differences were labelled as *P < 0.05; **P < 0.01; ***P < 0.001; ****P < 0.0001; n.s. = not significant. *P* values <0.05 was considered statistically significant.

## Results

### The phosphorylation of CHK2 at Thr68 is upregulated in OR CRC

**Fig. 1:**
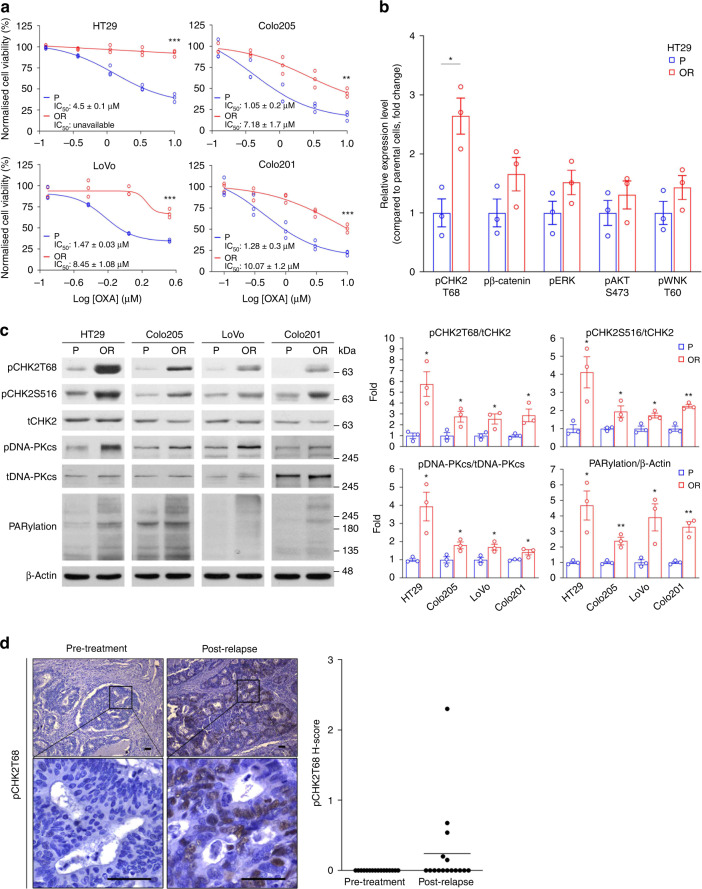
The level of phosho-CHK2 at T68 is upregulated in OR CRCs. **a** Viability of CRC oxaliplatin-resistant (OR) cell lines and their parental oxaliplatin-sensitive (P) counterparts after treatment with varying concentrations of oxaliplatin for 3 d. Data are mean ± SEM. The *P* values were determined by unpaired two-tailed Student’s *t* test, **P < 0.01, ***P < 0.001. *n* = 3. **b** The relative expression level of phosphorylated protein kinases as indicated. Data are mean ± SEM. The *P* values were determined by unpaired two-tailed Student’s *t* test, *P < 0.05. *n* = 3. **c** Representative western blots of a panel of CRC cell lines (left) and quantification analysis (right). Data are presented as the mean ± SEM. The *P* values were determined by unpaired two-tailed Student’s *t* test, *P < 0.05. *n* = 3. β-Actin served as a loading control. **d** Representative images of IHC analysis of phosphorylation of CHK2 at Thr68 (pCHK2T68) in sixteen pairs of pretreatment and post-relapse CRC samples from patients treated with the oxaliplatin-based regiment. Magnified images of the boxed areas are shown (left). Scale bar: 100 µm. The H-score of pCHK2T68 in sixteen pairs of pretreatment and postrelapse CRC samples from patients treated with an oxaliplatin-based regiment (right).

To explore the molecular mechanisms underlying oxaliplatin resistance in CRC, we performed drug resistance development assays. HT29, Colo201, Colo205 and LoVo CRC cell lines were chronically treated with stepwise increased concentrations of oxaliplatin. Cells were selected at each step until they formed colonies before moving to the next step. Throughout the 2–3 months of treatment, the oxaliplatin concentration was increased from 0.1 to 1.0 μM for the oxaliplatin-resistant (OR) group of cells. The sensitivity of cells to oxaliplatin was assessed by MTS-based cell viability (Fig. 1a) and clonogenic assays (Supplementary Fig. 1a). Next, we performed sphere formation assays in CRC cells to mimic cancerous properties and then identified the signalling nodes responsible for oxaliplatin resistance. Unlike other studies using two-dimensional (2D) culture, multicellular arrangements in 3D environments that establish a microenvironment with cell-cell contact and cell-extracellular matrix (ECM) contact are much more representative of their in vivo counterparts. HT29-OR and oxaliplatin-sensitive counterpart HT29-P cells were embedded in Matrigel containing 1.0 μM oxaliplatin and grown as tumour spheres. Lysates from these tumour spheres were collected and subjected to human phosphokinase array assays. Compared to HT29-P tumour spheres, we found that the phosphorylation levels of several protein kinases were upregulated in the lysates of HT29-OR tumour spheres. Among them, the phosphorylation of CHK2 at Thr68 (pCHK2T68) was the most pronounced (Fig. 1b and Supplementary Fig. 1b). The phosphorylation of threonine 68, one of the priming phosphorylation residues of CHK2, induces CHK2 dimerisation to promote the autophosphorylation of the kinase domain and T-loop at other residues, such as serine 516 (Ser516), leading to full CHK2 activation [17]. CHK2 was activated in OR CRC cells by examining the phosphorylation levels of CHK2 at both Thr68 and Ser516 in a panel of OR CRC cell lines and their oxaliplatin-sensitive counterparts (Fig. 1c). In pairs of pretreatment and post-relapse tumour samples from patients treated with oxaliplatin-based chemotherapy, we observed that the levels of pCHK2T68 were upregulated in five of the 16 pairs of post-relapse samples compared to their paired pretreatment samples (Fig. 1d). These results suggested that CHK2 activation is upregulated in oxaliplatin-resistant CRC cell lines and in ~30% of patients with acquired oxaliplatin-resistant CRC.

To explore the potential mechanism by which CHK2 mediates oxaliplatin resistance, we tested the activity of CHK2 upstream PIKKs, ATM, ATR and DNA-PKcs. We found that the basal levels of DNA-PKcs phosphorylation at Ser2056 (a marker for DSB-induced DNA-PKcs activation [15]) were increased in the four different OR CRC cell lines compared with their oxaliplatin-sensitive counterparts (Fig. 1c). The phosphorylation levels of ATR at Ser428 were upregulated in HT29-OR, Colo205-OR, and LoVo-OR cells. The levels of ATM phosphorylation were not significantly changed in these CRC cell lines (Supplementary Fig. 1c). Since CDC25C and p53 are downstream targets of CHK2, high levels of phospho-CDC25C at Ser216 were observed in HT29-OR and Colo205-OR cells. An elevated level of phospho-p53 at Ser20 was observed in HT29-OR cells (Supplementary Fig. 1c). We also found that HT29-OR and Colo205-OR cells have higher basal levels of γ-H2AX than their counterparts. PARP1 has been identified as a CHK2 substrate [23]. PARP1 promotes DNA repair by upregulating poly (ADP-ribosyl)ation (PARylation) of itself and target proteins [23, 26]. Antibodies against PARylation are used to determine the presence of PARP1-PARylated proteins, which present a high molecular weight smear in western blotting analysis. We observed that the levels of protein PARylation were upregulated in the four different OR CRC cell lines compared with their oxaliplatin-sensitive counterparts (Fig. 1c).

To monitor the kinetics of ATM, ATR, DNA-PKcs, CHK2 and CHK2 downstream effectors following oxaliplatin treatment, HT29-P and HT29-OR cells were treated with 10 μM oxaliplatin for 4 and 24 h. Afterwards, the cell lysates were collected and subjected to western blotting analysis. As shown in Supplementary Fig. 1d, in HT29-P cells, oxaliplatin treatment significantly induced the phosphorylation of ATM, ATR, CHK2 and p53 at the 4-h timepoint, and their phosphorylation intensity continued to increase until the 24-h timepoint. Moreover, the levels of phospho-DNA-PKcs, phospho-CDC25C and γ-H2AX were markedly upregulated at 24-h after oxaliplatin treatment. In contrast, HT29-OR cells showed higher basal phosphorylation levels of ATR, DNA-PKcs, CHK2, CDC25C, p53 and γ-H2AX, and oxaliplatin did not further induce their phosphorylation (Supplementary Fig. 1d). Taken together, we observed that [1] CHK2 and its upstream kinases, ATR and DNA-PKcs are constitutively activated in OR CRC cells, [2] the higher basal levels of protein PARylation indicate that higher PARP1 activity is in OR CRC cells, and [3] other CHK2 downstream effectors, phospho-p53, phospho-CDC25C and γ-H2AX, are upregulated in some OR CRC cells. These results suggest that [1] oxaliplatin activates CHK2 signalling in CRC cells and [2] OR CRC cells have upregulated CHK2 signalling, contributing to oxaliplatin resistance.

### HR is upregulated in OR CRC cells

**Fig. 2:**
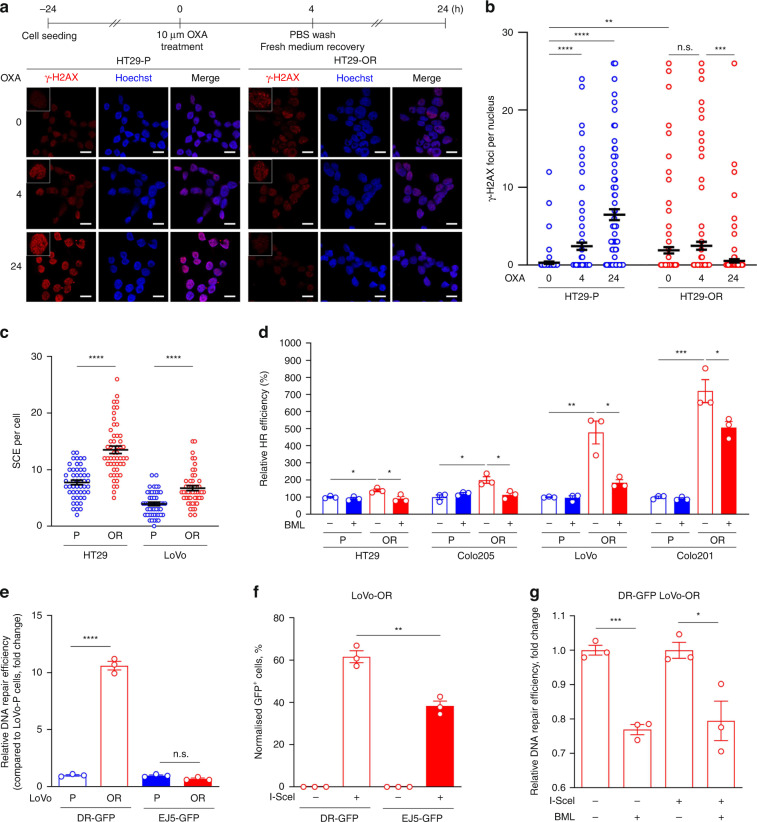
HR is upregulated in OR CRC cells. **a** A flowchart for oxaliplatin treatment schedule (top) and representative images of γ-H2AX foci were determined by fluorescence immunohistochemistry and confocal scanning laser microscopy (bottom). HT29-P and HT29-OR cells were treated with oxaliplatin for 4 h and withdrawal for 20 h. Scale bar: 50 μm. *n*  =  3. **b** Quantification analysis, related to (**a**), was performed in 100 cells. Data are mean ± SEM. The *P* values were determined by unpaired two-tailed Student’s *t* test, **P < 0.01, ***P < 0.001, ***P < 0.001, n.s. not significant. **c** Sister chromatid exchange (SCE) assay in CRC cells. Data are mean ± SEM. The *P* values were determined by unpaired two-tailed Student’s *t* test, ****P < 0.0001. SCE was scored in 50 metaphases of each cell line. **d** Homologous recombination assay of a panel of CRC cell lines treated with mock reagent or 10 μM of BML-277. Data are mean ± SEM. The *P* values were determined by unpaired two-tailed Student’s *t* test, *P < 0.05, **P < 0.01, ***P < 0.001. *n* = 3. **e** Relative DNA repair efficiency in the cells carrying pDR-GFP or pimEJ5-GFP. Data are mean ± SEM. The *P* values were determined by unpaired two-tailed Student’s *t* test, **P < 0.01, ****P < 0.0001. *n* = 3. **f** Percentage of GFP^+^ cells in LoVo-OR cells carrying pDR-GFP or pimEJ5-GFP. These cells were transiently transfected with pCAG or pCAG-I-SceI for 72 h before analysis were performed. Data are mean ± SEM. The *P* values were determined by unpaired two-tailed Student’s *t* test, **P < 0.01. *n* = 3. **g** Relative DNA repair efficiency in DR-GFP LoVo-OR. Cells carrying pCAG or expressing I-Sce1 were treated with mock reagent or 10 μM BML-277 for 72 h before analysis were performed. Data are mean ± SEM. The *P* values were determined by unpaired two-tailed Student’s *t* test, *P < 0.05, ***P < 0.001. *n* = 3.

To determine whether OR CRC cells have more efficient DNA repair ability than their oxaliplatin-sensitive counterparts, we performed DNA damage recovery assays. HT29-OR and HT29-P cells were treated with 10 μM oxaliplatin for 4 h. After removing oxaliplatin, the cells were allowed to recover in fresh medium for 20 h. The levels of DNA damage were indicated by the number of γ-H2AX foci, a marker for DNA lesions, using confocal microscopy (Fig. 2a, b). Oxaliplatin treatment induced γ-H2AX foci formation at the 4-h timepoint and continuously increased γ-H2AX foci after recovery for 20 h in HT29-P cells. In contrast, HT29-OR cells showed more pronounced γ-H2AX foci formation before treatment. Oxaliplatin treatment did not further induce γ-H2AX foci at the 4-h timepoint. However, compared with HT29-P cells, γ-H2AX foci were significantly reduced after recovery for 20-h in HT29-OR cells (Fig. 2a, b). These results suggest that HT29-OR cells have better DNA repair ability than HT29-P cells. Interestingly, we found that HT29-OR and LoVo-OR cells exhibited an elevated frequency of sister chromatid exchange (SCE) compared to their parental counterparts (Fig. 2c and Supplementary Fig. 2a). As SCE is the result of chromosome breaks and is subsequently repaired by HR, our results indicate that OR CRC cells encounter frequent chromosome breaks, and these DSBs can be repaired by HR. Consistent with the SCE results, we found that the protein levels of the HR proteins BRCA1 and BRCA2 were also elevated in OR CRC cells compared with their parental counterparts (Supplementary Fig. 2b).

Next, we evaluated the efficiency of HR repair in OR CRC cells and their counterparts by PCR-based HR assays. Cells were transfected with the dl-1 and dl-2 plasmid mix or the control plasmid. Twenty-four hours after transfection, the cellular DNA was isolated and subjected to qPCR analysis to evaluate HR efficiency. As shown in Fig. 2d, the levels of relative HR activity were increased by approximately 1.4-fold in HT29-OR cells, 2.0-fold in Colo205-OR cells, 4.8-fold in LoVo-OR cells, and 7.2-fold in Colo201-OR cells compared to their oxaliplatin-sensitive counterparts. BML-277 is a CHK2 kinase inhibitor. Interestingly, BML-277 treatment significantly reduced HR activity in these OR CRC cell lines, with an ~30% reduction in HT29-OR cells, a 40% reduction in Colo205-OR cells, a 60% reduction in LoVo-OR cells, and a 30% reduction in Colo201-OR cells. These results suggest that CHK2 activity is required for HR.

In addition, we performed reporter-based DSB repair assays to determine the HR activity in LoVo and LoVo-OR cells. pDR-GFP (Direct Repeat GFP) [27], which contains two incomplete GFP cassettes, is stably expressed in the cells. The first GFP cassette contains a promoter and the I-SceI restriction site with a premature stop codon, and the second cassette has an intact coding sequence but lacks a promoter. A DSB is created in the first cassette by introducing I-SceI endonuclease and is repaired by HR using the second cassette as a template to create a promoter-driven intact GFP gene. The HR efficiency can be determined by the percentage of GFP^+^ cells using flow cytometry (Supplementary Fig. 2c). After generating DR-GFP stable clones by puromycin selection, we found that a small population of GFP^+^ cells was observed in these stable clones even before the introduction of I-SceI plasmids, especially in LoVo-OR cells. The percentage of GFP^+^ cells in LoVo-OR cells was approximately tenfold higher than that in LoVo-P cells (0.93 ± 0.05% in LoVo-OR cells vs. 0.08 ± 0.02% in LoVo-P cells, *P* < 0.0001) (Fig. 2e and Supplementary Fig. 2d).

Since DSBs could also be repaired by NHEJ, we then generated EJ5-GFP (end-joining GFP) stable clones in LoVo and LoVo-OR cells. pEJ5-GFP [28] contains a promoter that is separated from a GFP cassette by a puromycin resistance (*puroR*) gene flanked by two I-SceI sites in the same orientation. The I-SceI-induced DSBs in the two I-SceI sites are repaired by NHEJ to join the promoter and the GFP cassette together, resulting in GFP expression. The NHEJ efficiency was determined by the percentage of GFP^+^ cells using flow cytometry (Supplementary Fig. 2c). In contrast to DR-GFP, EJ5-GFP stable clones derived from LoVo-P or LoVo-OR cells did not show significant differences in the percentage of GFP^+^ cells (Fig. 2e and Supplementary Fig. 2d). These results indicate that LoVo-OR cells have higher spontaneous HR activity than LoVo-P cells. When I-SceI endonuclease was induced into these DR-GFP and EJ5-GFP stable clones, I-SceI induced more GFP^+^ cells in LoVo-OR cells carrying DR-GFP than EJ5-GFP (61.6 ± 4.85% vs. 38.24 ± 4.13%, *P* = 0.003), indicating that HR repair could be more dominant than NHEJ in LoVo-OR cells (Fig. 2f).

In addition, BML-277 treatment significantly reduced HR efficiency in the absence or presence of I-SceI in DR-GFP LoVo-OR stable clones (Fig. 2g). These results support that OR CRC cells encounter more frequent replication stress and chromosome breaks; however, upregulated HR activity allows OR CRC cells to overcome replication stress and repair DSBs. Moreover, the results of BML-277 treatment also suggest that CHK2 activity is critical for HR in CRC cells.

### CHK2 promotes PARylation and facilitates DNA repair

**Fig. 3:**
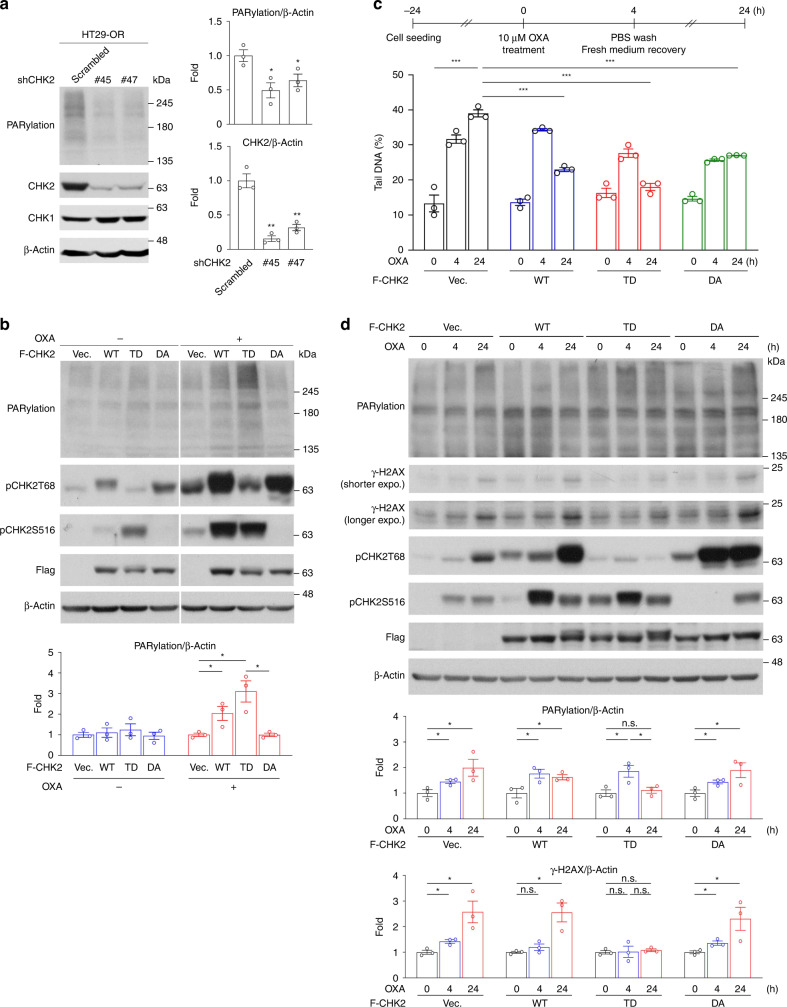
CHK2 promotes PARylation and facilitates DNA repair. **a** Representative western blots of HT29-OR cells that were stably expressing either a scrambled shRNA or shRNAs specific for CHK2 (#45 and #47) (left) and quantification analysis (right). Data are mean ± SEM. The *P* values were determined by unpaired two-tailed Student’s *t* test, *P < 0.05, **P < 0.01. *n* = 3. β-Actin served as a loading control. **b** Representative western blots of oxaliplatin treatment experiments on HT29-P cells stably carrying the empty vector or expressing F-CHK2 constructs as indicated (top) and quantification analysis (bottom). Data are mean ± SEM. The *P* values were determined by unpaired two-tailed Student’s *t* test, *P < 0.05. *n* = 3. β-Actin served as a loading control. **c** Comet assay for oxaliplatin treatment experiments on HT29-P cells stably carrying the empty vector or expressing F-CHK2 constructs as indicated. Top: a flowchart for oxaliplatin treatment schedule. Bottom: percentage of tail DNA. Data are mean ± SEM. The *P* values were determined by unpaired two-tailed Student’s *t* test, ***P < 0.001. *n* = 3. **d** Representative western blots of oxaliplatin treatment experiments from (**c**, top) and quantification analysis (bottom). Data are mean ± SEM. The *P* values were determined by unpaired two-tailed Student’s *t* test, *P < 0.05, n.s. not significant. *n* = 3. β-Actin served as a loading control.

PARP1 has been suggested to interact with and be phosphorylated by CHK2 in response to oxidative DNA damage [23]. To determine whether CHK2 mediates the upregulation of protein PARylation in OR CRC cells, shRNAs specific for *CHK2* were used to downregulation the expression of CHK2. Two independent CHK2-specific shRNAs, #45 and #47, were tested to avoid potential off-target effects of shRNAs. We found that HT29-OR cells expressing either *CHK2* shRNA had reduced CHK2 expression and protein PARylation compared to scrambled shRNA controls (Fig. 3a), suggesting CHK2 knockdown inactivates PARP1. Next, we examined whether the activation of PARP1 is dependent on CHK2 activity. Flag-tagged CHK2 (F-CHK2 hereafter) constructs, including the wild-type (WT), a phosphomimetic mutant in which T68 residue was substituted with Asp (TD), and a dominant negative kinase-dead mutation in which Asp347 was replaced by Ala (DA), were stably expressed in HT29-P cells. We found that oxaliplatin-induced protein PARylation was promoted in cells expressing F-CHK2-WT and was further enhanced in F-CHK2-TD. Such a promotion effect was not observed in cells expressing F-CHK2-DA (Fig. 3b). These results showed that CHK2 activity is required for PARP1 activation.

The influence of CHK2 activity on oxaliplatin-induced DNA damage repair was determined using comet assays. HT29-P cells stably expressing empty vector, F-CHK2-WT, or CHK2 mutants were treated with 10 μM oxaliplatin for 4 h before withdrawal treatment for 20 h (Fig. 3c). Cells collected at different time points were subjected to comet assays. The percentage of comet DNA in the tail was measured after oxaliplatin treatment and had a mean value close to ~30%, indicating that 4 h oxaliplatin treatment induces DNA damage compared to mock treatment. Oxaliplatin-induced DNA strand breaks were observed 20 h after oxaliplatin withdrawal in HT29-P cells expressing empty vector and were modestly reduced in the expression of wild-type CHK2. DNA repair was accelerated in cells expressing the phosphorylation mimic F-CHK2-TD but not in DA (Fig. 3c and Supplementary Fig. 3). Next, the cell lysates were collected and subjected for western blotting analysis. We found that oxaliplatin-induced protein PARylation was sustained in cells expressing empty vector, F-CHK2-WT, and DA after oxaliplatin removal for 20 h, whereas the levels of PARylation in cells expressing F-CHK2-TD were reduced to basal levels within 20 h after oxaliplatin withdrawal (Fig. 3d). Consistently, the treatment of oxaliplatin continuously stimulated γ-H2AX expression in the cells expressing empty vector, F-CHK2-WT and DA even oxaliplatin withdrawal for 20 h. The reduced levels of oxaliplatin-induced γ-H2AX was observed in the cells expressing F-CHK2-TD after oxaliplatin withdrawal (Fig. 3d), indicating that the expression of CHK2-TD reduces oxaliplatin-induced DSBs after oxaliplatin withdrawal. These results suggested that CHK2 activity plays a crucial role in oxaliplatin-induced DNA damage repair.

### Oxaliplatin induces the CHK2/PARP1 interaction

**Fig. 4:**
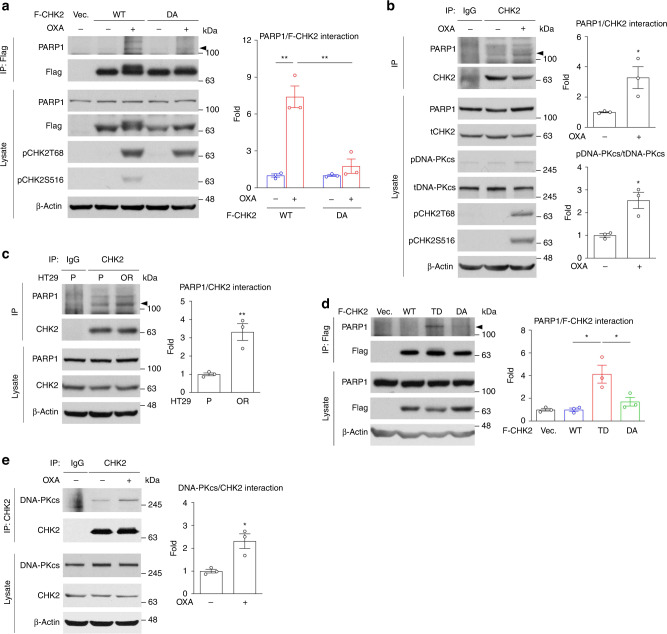
Oxaliplatin induces the CHK2/PARP1 interaction. **a** Representative western blots of PARP1 coimmunoprecipitated with anti-FLAG M2 affinity gel (left) and quantification analysis (right). Data are mean ± SEM. The *P* values were determined by unpaired two-tailed Student’s *t* test, **P < 0.01. HT29-P cells stably expressing F-CHK2 constructs were mock-treated (−) or treated with oxaliplatin (+), cell lysates were prepared and immunoprecipitated with anti-FLAG M2 affinity gel. Cells expressing an empty vector were served as a control. β-Actin served as a loading control. *n* = 3. **b** Representative western blots of PARP1 coimmunoprecipitated with anti-CHK2 antibody-conjugated agarose (left) and quantification analysis (right). Data are mean ± SEM. The *P* values were determined by unpaired two-tailed Student’s *t* test, *P < 0.05. HT29-P cells were mock-treated (−) or treated with oxaliplatin (+), cell lysates were prepared and immunoprecipitated with anti-CHK2 antibody-conjugated agarose. Mouse IgG antibodies served as an IP control and β-Actin served as a loading control. *n* = 3. **c** Representative western blots of PARP1 coimmunoprecipitated with anti-CHK2 antibody-conjugated agarose (left) and quantification analysis (right). Data are mean ± SEM. The *P* values were determined by unpaired two-tailed Student’s *t* test, **P < 0.01. Lysates from HT29-P and HT29-OR cells were prepared and immunoprecipitated with anti-CHK2 antibody-conjugated agarose. Mouse IgG antibodies served as an IP control and β-Actin served as a loading control. *n* = 3. **d** Representative western blots of PARP1 coimmunoprecipitated with anti-FLAG M2 affinity gel (left) and quantification analysis (right). Data are mean ± SEM. The *P* values were determined by unpaired two-tailed Student’s *t* test, *P < 0.05. Cell lysates of HT29-P cells stably expressing F-CHK2 constructs were prepared and immunoprecipitated with anti-FLAG M2 affinity gel. Cells expressing an empty vector were served as a control. β-Actin served as a loading control. *n* = 3. **e** Representative western blots of DNA-PKcs coimmunoprecipitated with anti-CHK2 antibody-conjugated agarose (left) and quantification analysis (right). Data are mean ± SEM. The P values were determined by unpaired two-tailed Student’s t test, *P < 0.05. HT29-P cells were mock-treated (−) or treated with oxaliplatin (+), cell lysates were prepared and immunoprecipitated with anti-CHK2 antibody-conjugated agarose. Mouse IgG antibodies served as an IP control and β-Actin served as a loading control. *n* = 3.

Next, we carried out coimmunoprecipitation (co-IP) assays to address whether CHK2/PARP1 forms a complex in response to oxaliplatin. Cell lysates collected from HT29-P cells expressing F-CHK2-WT or empty vector treated with oxaliplatin were subjected to immunoprecipitation with an anti-FLAG M2 affinity agarose gel. When analysed by western blotting, we found that PARP1 was barely associated with the anti-FLAG immunoprecipitation complex in cells expressing F-CHK2-WT but was greatly enhanced in response to oxaliplatin treatment (Fig. 4a). Moreover, the endogenous CHK2/PARP1 interaction was promoted by oxaliplatin treatment (Fig. 4b) and was enhanced in HT29-OR cells (Fig. 4c) when endogenous CHK2 was immunoprecipitated with anti-CHK2 antibody-conjugated agarose. Notably, the CHK2/PARP1 interaction was increased in cells expressing F-CHK2-TD but not in cells expressing F-CHK2-WT or F-CHK2-DA or carrying an empty vector (Fig. 4d). The oxaliplatin-induced CHK2/PARP1 interaction was disrupted in cells expressing F-CHK2-DA, showing the CHK2-PARP1 interaction is dependent on CHK2 kinase activity (Fig. 4a). Similar results were suggested by a previous report that CHK2 interacts with and activates PARP1 in response to oxidative stress [23].

The DNA-PKcs activity has been associated with phosphorylation and activation of CHK2 [19, 20], but the interaction of DNA-PKcs and CHK2 in response to oxaliplatin has not been revealed yet. We found that CHK2 was able to precipitate DNA-PKcs endogenously and oxaliplatin enhanced this interaction in HT29-P cells (Fig. 4e). We showed that oxaliplatin induces the formation of CHK2/PARP1 and DNA-PKcs/CHK2 complexes, suggesting that oxaliplatin-indued DNA-PKcs/CHK2/PARP1 complex could be one of the mechanisms that activate CHK2/PARP1 signalling in CRC cells.

### The kinase activity of CHK2 is critical for overcoming oxaliplatin

**Fig. 5:**
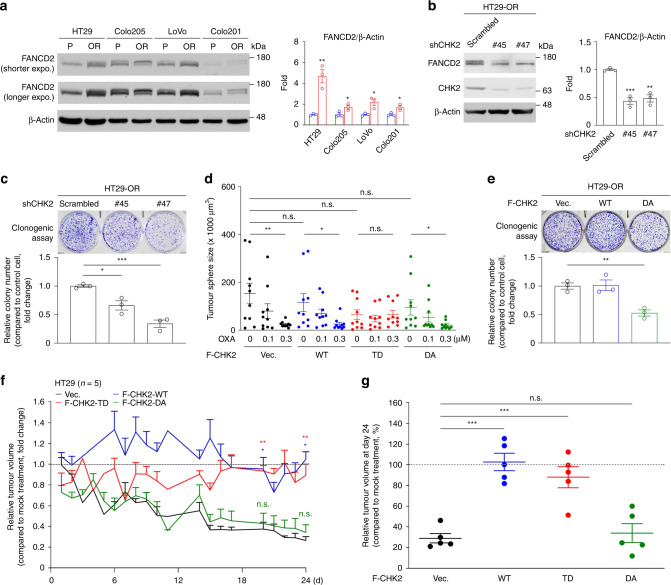
CHK2 activity is critical for overcoming oxaliplatin. **a** Representative western blots of a panel of CRC cell lines (left) and quantification analysis (right). Data are mean ± SEM. The *P* values were determined by unpaired two-tailed Student’s *t* test, *P < 0.05, **P < 0.01. β-Actin served as a loading control. *n* = 3. **b** Representative western blots of HT29-OR cells that were stably expressing either a scrambled shRNA or shRNAs specific for CHK2 (#45 and #47) (left) and quantification analysis (right). Data are mean ± SEM. The *P* values were determined by unpaired two-tailed Student’s *t* test, **P < 0.01, ***P < 0.001. β-Actin served as a loading control. *n* = 3. **c** Representative images (top) and relative colony numbers (bottom) of clonogenic growth assays in CHK2-knockdown cells described in (**b**). Three thousand cells were seeded in six-well plates for 14 d. Data are mean ± SEM. The *P* values were determined by unpaired two-tailed Student’s *t* test, *P < 0.05, ***P < 0.001. *n* = 3. **d** The size of tumour spheres in anchorage-independent growth assay. HT29-P cells stably carrying the empty vector or expressing F-CHK2 constructs were grown as tumour spheres in the presence of varying concentrations of oxaliplatin for 3 wk. Data are mean ± SEM. The *P* values were determined by unpaired two-tailed Student’s *t* test, *P < 0.05, **P < 0.01. n.s. not significant. *n* = 3. **e** Representative images (top) and relative colony numbers (bottom) of clonogenic growth assays in HT29-OR cells stably carrying the empty vector or expressing F-CHK2 constructs. Three thousand cells were seeded in six-well plates for 14 d. Data are mean ± SEM. The *P* values were determined by unpaired two-tailed Student’s *t* test, **P < 0.01. *n* = 3. **f** The curves of relative tumour volume in the mice bearing xenograft tumours derived from HT29 cells stably carrying the empty vector or expressing F-CHK2 constructs. Relative tumour volume was calculated by oxaliplatin-treated tumour volume/mock-treated tumour volume. Data are mean ± SEM. The *P* values were determined by unpaired two-tailed Student’s *t* test, *P < 0.05, **P < 0.01. n.s. not significant. Mock treatment, *n* = 5; oxaliplatin treatment, *n* = 5. **g** Relative tumour volume at d 24 as described in (**f**), was calculated by oxaliplatin-treated volume/mock-treated volume × 100%. Data are mean ± SEM. The *P* values were determined by unpaired two-tailed Student’s *t* test, ***P < 0.001. n.s. not significant.

The CHK2/PARP1 pathway was upregulated in OR CRC cells. A clinical PARP1 inhibitor, Olaparib, was employed to test the inhibitory effects on OR CRC. We found that Olaparib decreased the levels of protein PARylation in HT29-OR cells but did not suppress the proliferation of a panel of OR CRC cells in MTS assays (Supplementary Fig. 4a, b). Olaparib is effective to treat HR-defective cancer cells, but not HR-proficient cancer cells [29]. Since OR CRC cells have proficient HR, it explains that Olaparib is not toxic to these OR CRC cells. The Fanconi anaemia (FA)/BRCA pathway has been suggested to play a crucial role in ICLs-indued DNA damage repair [30, 31] and contribute to chemoresistant phenotypes in head and neck squamous cell carcinomas [32] and breast cancer cells [33]. We then checked the monoubiquitination levels of FANCD2, a key factor for FA pathway activation, and found that the levels of FANCD2 monoubiquitination was upregulated in OR CRC cell lines compared to their parental counterparts (Fig. 5a). More, Olaparib treatment did not reduce the level of FANCD2 monoubiquitination in HT29-OR cells (Supplementary Fig. 4a), suggesting that PARP1 PARylation activity is not involved in FANCD2 monoubiquitination process. CHK2 knockdown reduced the levels of FANCD2 monoubiquitination in HT29-OR cells (Fig. 5b), showing CHK2 is critical for FA pathway activation. These results implied that CHK2 regulates the activity of both PARP1 and FA pathways to facilitate oxaliplatin-induced DNA repair.

Next, we carried out clonogenic cell growth assays to determine whether CHK2 activity is critical for cell survival in presence of oxaliplatin. Firstly, we observed that CHK2 silencing inhibited colony growth in HT29-OR cells (Fig. 5c). The gain-of-function experiments indicated that oxaliplatin suppressed the colony size of HT29-P cells carrying empty vector and expressing F-CHK2-WT but not in cells expressing F-CHK2-TD in anchorage-independent growth assays. Moreover, the cells expressing F-CHK2-DA were sensitive to oxaliplatin treatment (Fig. 5d and Supplementary Fig. 4c). In addition, the expression of F-CHK2-DA decreased the colony number of HT29-OR cells in clonogenic cell growth assays (Fig. 5e). These results indicated that the kinase activity of CHK2 plays an important role in overcoming the cytotoxic effects of oxaliplatin.

Next, we determined the impact of CHK2 activity on the development of oxaliplatin resistance in xenograft tumour models. HT29-P cells carrying an empty vector or expressing wild-type F-CHK2 and its mutants were grown as xenograft tumours. These tumour-bearing mice were administered intraperitoneally with a mock agent and oxaliplatin at 5 mg/kg once weekly for 3 weeks. We observed that the administration of oxaliplatin reduced the tumour volume when HT29-P cells were carrying an empty vector or expressing F-CHK2-DA, whereas the size of tumours raised from the cells expressing F-CHK2-WT or F-CHK2-TD was sustained (Fig. 5f and Supplementary Fig. 4d). At the end of oxaliplatin treatment, the size of tumours carrying a control vector was reduced up to 70.9 ± 4.5% but was modestly reduced when the tumours expressed F-CHK2-WT or F-CHK2-TD. Additionally, tumours expressing F-CHK2-DA had decreased volume up to 66 ± 9.1% (Fig. 5g). The body weights of these animals were comparable during the treatment courses (Supplementary Fig. 4e). These results showed that phosphorylation of CHK2 at T68 is crucial for developing oxaliplatin resistance.

### BML-277 suppresses the growth of OR CRC in vitro and in vivo

**Fig. 6:**
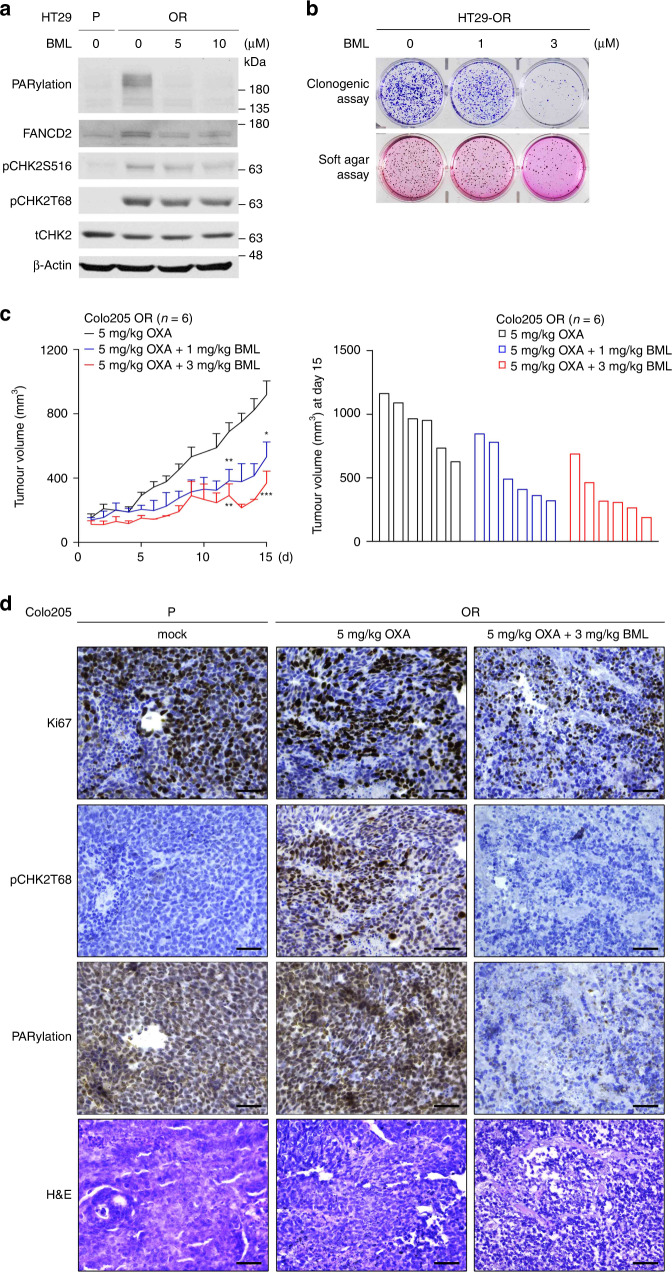
BML-277 suppresses the growth of OR CRC in vitro and in vivo. **a** Representative western blots of BML-277 treatment experiments on HT29-OR cells. β-Actin served as a loading control. *n* = 3. **b** Representative images of clonogenic growth (top) and soft agar colony formation (bottom) of BML-277 treatment experiments on HT29-OR cells. *n* = 3. **c** Quantification of tumour growth curves of tumour size (left) and the waterfall plats of tumour size at d 15 (right) in the mice bearing xenograft tumours originating from Colo205-P and Colo205-OR cells treated with 5 mg/kg oxaliplatin once per week (*n* = 6), 5 mg/kg oxaliplatin once per week combined with 1 mg/kg BML-277 three times weekly (*n* = 6), or 5 mg/kg oxaliplatin once per week combined with 3 mg/kg BML-277 three times weekly (*n* = 6) by intraperitoneal injection. Data are mean ± SEM. The *P* values were determined by unpaired two-tailed Student’s *t* test, *P < 0.05, **P < 0.01, ***P < 0.001. **d** Representative images of H&E staining and IHC analysis of pCHK2T68, PARylation and Ki67 levels in xenograft tumour samples displayed in (**c**). Scale bar: 50 μm. *n* = 6.

To evaluate whether pharmacological inhibition of CHK2 could attenuate the growth of OR CRC cells, BML-277, an ATP-competitive inhibitor of CHK2, was employed. As a result, BML-277 attenuated CHK2 activity, protein PARylation and FANCD2 monoubiquitination in HT29-OR cells (Fig. 6a and Supplementary Fig. 5a). BML-277 treatment suppressed colony formation in clonogenic and anchorage-independent cell growth assays in OR CRC cells (Fig. 6b and Supplementary Fig. 5b). These results further supported that the activity of CHK2 is crucial for OR CRC cells to overcome the anti-proliferation effect of oxaliplatin.

Finally, we evaluated the antitumor activity of BML-277 in suppressing the tumour growth of OR CRC cells. Oxaliplatin sensitivity assays were performed in xenograft tumour models using oxaliplatin-resistant Colo205-OR cells and its oxaliplatin-sensitive counterpart Colo205-P cells. Oxaliplatin administration at 5 mg/kg once weekly for 2 weeks significantly suppressed the growth of tumours raised from Colo205-P cells but not from Colo205-OR cells (Supplementary Fig. 5c). Next, the Colo205-OR tumour-bearing mice were grouped and administered intraperitoneally with oxaliplatin at 5 mg/kg once weekly for 2 weeks, oxaliplatin at 5 mg/kg once weekly combined with 1 mg/kg BML-277 thrice weekly for 2 weeks, or oxaliplatin at 5 mg/kg once weekly combined with 3 mg/kg BML-277 thrice weekly for 2 weeks. The results indicated that the presence of BML-277 significantly suppressed the Colo205-OR tumour growth (Fig. 6c). The body weights of these animals were comparable during the treatment courses (Supplementary Fig. 5d). These Colo205-OR tumours were then collected and subjected to IHC analysis, showing that the administration of BML-277 eliminated actively proliferating tumour cells and reduced the levels of pCHK2T68 and PARylation (Fig. 6d). Our results supported that therapeutic benefit may be achieved by means of the CHK2 inhibitor BML-277 in oxaliplatin-resistant colorectal cancer.

## Discussion

In this study, we identified that CHK2 phosphorylation at T68 (pCHK2T68) was the most pronounced kinase among the protein kinases in HT29-OR cells using human a phosphokinase array. We further revealed that a panel of OR CRC cell lines and ~30% of oxaliplatin-resistant CRC samples derived from patients showed elevated levels of pCHK2T68. In addition to pCHK2T68, these OR CRC cells exhibited elevated CHK2 upstream PIKKs and downstream effector activation, protein PARylation, SCE frequency, and HR activity compared to their parental counterparts. A phosphorylation mimicking CHK2 mutant, CHK2T68D, but not a kinase-dead CHK2 mutant, CHK2D347A, promoted DNA repair, cell colony formation, and xenograft tumour growth in the presence of oxaliplatin. We further identified that CHK2 interacted with PARP1 and promoted protein PARylation and FANCD2 monoubiquitination. A CHK2 inhibitor, BML-277, reduced HR activity, FANCD2 monoubiquitination, and protein PARylation, resulting in repression of OR CRC cell growth in vitro and in vivo. Our findings suggest that CHK2 activity is critical for DNA repair and oxaliplatin resistance in CRC cells (Supplementary Fig. 6).

Our results show that OR CRC exhibited high phosphorylation levels of CHK2 and its upstream PIKKs and downstream effectors, indicating that the OR CRC cells experienced more frequent replication stress and chromosomal breaks. We observed that the OR CRC cells exhibited higher phosphorylation levels of ATR, DNA-PKcs, and H2AX than their oxaliplatin-sensitive counterparts. ATR is activated in response to replication stress. DNA-PKcs is phosphorylated by ATR during replication stress, in addition to its role in response to DSBs and NHEJ. γ-H2AX is a general DNA damage marker, that indicates stalled replication forks, single-strand DNA breaks (SSBs), and double-strand DNA breaks. These PIKKs phosphorylate CHK2, resulting in CHK2 activation and the signal is transduced to the downstream effectors, p53 and CDC25C. These results suggest that OR CRC cells experience replication stress. Interestingly, we also found that HT29-OR and LoVo-OR exhibited elevated sister chromatid exchanges (SCEs) compared to their counterparts (Fig. 2c and Supplementary Fig. 2a). SCE is the result of chromosome breaks and is repaired by HR. High levels of ATR, DNA-PKcs, CHK2, and H2AX phosphorylations and SCE indicate that OR CRC cells encounter more frequent DSBs, possibly resulting from replication stress. However, these DSBs can be efficiently repaired by HR in these OR CRC cells. Using a PCR-based HR assay (Fig. 2d) or DR-GFP HR assay (Fig. 2e, f), OR CRC cells exhibited better HR efficiency than their counterparts. Moreover, CHK2 activates HR and promotes FANCD2 monoubiquitination, a key step in the FA repair pathway. These results are in line with our conclusion that OR CRC cells encounter more frequent replication stress and chromosome breaks; however, OR CRC cells have the ability to overcome replication stress and repair DSBs through CHK2-mediated FA and HR repair pathways.

Studies suggest that PARP1 is recruited and activated by DSBs. Activated PARP1 promotes protein PARylation on itself or a variety of target proteins, which is an important process for DNA repair [34]. Here, we identified that CHK2 interacts with PARP1 and that oxaliplatin treatment enhanced this interaction. Interestingly, OR CRC cells with elevated levels of CHK2 phosphorylation and protein PARylation also showed an upregulated CHK2/PARP1 interaction. CHK2 knockdown or inhibition reduced the levels of protein PARylation, suggesting that CHK2 kinase activity regulates PARP1 PARylation activity. Consistent with our findings, a recent study demonstrated that CHK2 interacts with and phosphorylates PARP1 at three major sites, Thr420, Thr622 and Thr656, stimulating the PARylation activity of PARP1 in response to oxidative DNA damage [23]. PARP1 is also activated and phosphorylated by the receptor tyrosine kinase c-Met at Tyr907 [35] and c-Jun-N-terminal kinase 1 (JNK1) [36] under oxidative stress. Although oxaliplatin-induced ICLs are different from H_2_O_2_-induced DNA damage, both induce replication stress. CHK2 could interact and phosphorylate PARP1, resulting in the stimulation of PARylation of PARP1; however, we still cannot rule out the possibility that other kinases, such as c-Met and JNK1, or other unidentified molecules mediate PARP1 activation.

PARP1 also participates in base excision repair (BER) and nucleotide excision repair (NER) pathways [37]. The elevated protein PARylation in OR CRC cells implies that OR CRC cells could acquire enhanced BER/NER pathways. Although BER/NER pathways were not tested in this study, our DNA damage recovery assay indeed revealed that OR CRC cells acquired more efficient DNA repair ability, leading to oxaliplatin resistance. In addition, previous studies have demonstrated the importance of NER in chemotherapy. For example, ERCC1 expression levels have an inverse correlation with either response to platinum-based adjuvant chemotherapy or survival in patients with ovarian cancer, colorectal cancer, and non-small cell lung cancer (NSCLC) [38–40]. The ERCC1 protein levels or *ERCC1* polymorphisms in CRC also correlated with the response to oxaliplatin-based chemotherapy and survival. These studies suggest that ERCC1 could serve as a prognostic biomarker and as a potential therapeutic target [39, 41–43]. Small molecule inhibitors targeting the ERCC1–XPF complex by interrupting the complexes of ERCC1–XPA or ERCC1–XPF or by suppressing the endonuclease activity of XPF have been developed [44]. Our study revealed that CHK2 activates PARP1, also implying the enhancement of BER/NER pathways.

The FA pathway has been associated with PARP inhibitor resistance in head and neck cancers [32], and the overexpression of FANCD2 confers PARP inhibitor resistance in BRCA1/2 mutant breast cancer cells [33]. FANCD2 monoubiquitination at K561 is the key step and is an indicator of FA signalling activation; however, the mechanics for precise FANCD2 monoubiquitination remain elusive [45]. ATR kinase-mediated phosphorylation of FANCI (a binding partner of FANCD2) promotes FANCI/ FANCD2 complex formation, which is required for FANCD2 monoubiquitination and prevents USP1-UAF1-mediated deubiquitination [46]. Moreover, the levels of FANCD2 monoubiquitination are proposed to be regulated by casein kinase 2 [47]. Our studies show that the knockdown or inhibition of CHK2 decreases FANCD2 monoubiquitination in OR CRC cells (Figs. 5b, 6a and Supplementary Fig. 5a), indicating that CHK2 kinase activity could regulate FANCD2 monoubiquitination. It is, however, not clear whether CHK2 interacts and phosphorylates FANCD2. Further investigation is needed in the future.

The in vitro cell and in vivo xenograft tumour models demonstrated that [1] cells expressing CHK2T68D grown as tumour spheres or xenograft tumours were less sensitive to oxaliplatin and [2] treatment with BML-277 suppressed OR cells grown as colonies, tumour spheres, and xenograft tumours, supporting the idea that the activation of CHK2 signalling is critical for overcoming oxaliplatin and is associated with the development of oxaliplatin resistance in colorectal cancer. Our study revealed not only the association between the levels of pCHK2T68 and oxaliplatin resistance in CRC but also the molecular mechanism by which CHK2 signalling participates in DNA damage repair, CHK2/PARP1 interaction, cell colony formation, and xenograft tumour growth in the presence of oxaliplatin. BML-277, a CHK2 inhibitor, reduced protein PARylation, FANCD2 monoubiquitination, HR efficiency, and OR CRC cell growth in vitro and in vivo. These results suggest that CHK2 is a potential therapeutic target for OR CRC.

## Supplementary information

Supplementary information

Supplementary figure 1

Supplementary figure 2

Supplementary figure 3

Supplementary figure 4

Supplementary figure 5

Supplementary figure 6

Non-Cropped WB Images

## Data Availability

All materials are available upon reasonable request to chshen@nhri.edu.tw.
