# Supplementary material for: CHK2 activation contributes to the development of oxaliplatin resistance in colorectal cancer
Source: Br J Cancer. 2022 Aug 23;127(9):1615–28. doi: 10.1038/s41416-022-01946-9 (PMC9596403; doi:10.1038/s41416-022-01946-9)
Supplement: Supplementary file 1 — Supplementary information [file 41416_2022_1946_MOESM1_ESM.docx]

**Supplementary Materials and Methods**

**Reagents and antibodies**

Anti-phospho-CHK2 (pThr68) (pCHK2T68, #2197, western blotting 1:1000, immunohistochemistry 1:100), anti-phospho-CHK2 (pSer516) (#2669, western blotting 1:1000), anti-ATM (#2873, western blotting 1:1000), anti-CDC25C (#9555, western blotting 1:1000), anti-CHK1 (#2360, western blotting 1:1000), anti-phospho-p53 (pSer20) (#9287, western blotting 1:1000), anti-p53 (#2527, western blotting 1:1000), and anti-Ki67 (D2H10, immunohistochemistry 1:200) were from Cell Signaling Technology. Anti-CHK2 (05-649SP, western blotting 1:1000), anti-Poly ADP-ribose (clone 10H, western blotting 1:1000, immunohistochemistry 1:200), anti-γ-H2AX (#05-636, immunofluorescence 1:1000), and anti-phospho-ATM (05-740SP, western blotting 1:1000) were purchased from Millipore. Anti-phospho-DNA-PKcs (ab124918, western blotting 1:1000), anti-BRCA2 (ab123491, western blotting 1:1000), and anti-phospho-CDC25C (ab156574, western blotting 1:1000) were from Abcam. Anti-DNA-PKcs (sc-5282, western blotting 1:1000), anti-PARP1 (sc-8007, western blotting 1:1000), anti-FANCD2 (sc-20022, western blotting 1:1000), anti-α-Tubulin (sc-5286, western blotting 1:1000), and anti-CHK2 antibody (A-12)-conjugated agarose were purchased from Santa Cruz. Anti-Flag M2 (western blotting 1:1000) antibodies and anti-FLAG M2 affinity gel were from Sigma-Aldrich. Anti-BRCA1 (GTX50557, western blotting 1:500) were purchased from GeneTex. Oxaliplatin, BML-277, and Olaparib were purchased from Selleck Chemicals.

**Human phospho-kinase array**

Tumor spheres were collected by centrifugation, washed by ice-cold PBS, and lysed by RIPA buffer (Thermo Fisher Scientific) containing 10 mM β-glycerophosphate, 10 nM calyculin A, 1 mM Na_3_VO_4_ and protease inhibitors. The lysates were incubated with Proteome Profiler Human Phospho-Kinase Array Kit (R&D systems, Abingdon, UK) as manufacturer’s instructions. The intensity of dots was quantified and analysis using NIH ImageJ software.

**Constructs**

Full‐length CHK2 cDNA was a gift from Stephen Elledge (Addgene plasmid # 41901)(1). A Flag tag was added on the N-terminal of CHK2 and was cloned into pBabe-Puro vector using PCR-based subcloning. CHK2 mutants were generated using PCR-based mutagenesis. For knockdown experiments, pLKO constructs containing shRNAs against CHK2 (shCHK2#45: TRCN0000039945; shTRPM1#47: TRCN0000039947) were purchased from the National RNAi Core Facility at Academia Sinica (Taipei, Taiwan). All constructs were verified by sequencing.

For the reporter-based HR repair and NHEJ assays, pDR-GFP (Direct Repeat-GFP) was a gift from Maria Jasin (#26475, Addgene) (4) and pimEJ5-GFP was a gift from Jeremy Stark (#44026, Addgene) (5).

**Western blotting and co-immunoprecipitation.**

Western blotting and immunoprecipitation were performed as previously described (2). Briefly, cell lysates were prepared using lysis buffer containing Tris-HCl pH 7.4, 150 mM NaCl, 1% NP-40, 1 mM EDTA, 50 mM NaF, 10 mM β-glycerophosphate, 10 nM calyculin A, 1 mM Na_3_VO_4_ and protease inhibitors, and normalized by protein concentrations using the Bradford method (Bio-Rad). For western blotting, cell lysates were boiled in Laemmli sample buffer and separated on SDS-PAGE and transferred to Immobilon-P PVDF Membrane (Sigma-Aldrich). The membranes were blocked in TBST containing 5% of nonfat milk, incubated with primary antibodies based on the manufacturer’s instructions, followed by incubation with horseradish peroxidase-conjugated goat anti-rabbit or anti-mouse IgG (Thermo Fisher) and enhanced chemiluminescence detection (Sigma-Aldrich).

For co-immunoprecipitation, cell lysates were incubated with anti-FLAG M2 affinity agarose gel, or anit-CHK2 conjugated agarose at 4°C overnight. Beads were washed three times with lysis buffer and boiled in Laemmli sample buffer, and immune complexes were analyzed by SDS-PAGE and western blotting.

**MTS, clonogenic growth, and anchorage-independent cell growth assays**

MTS, clonogenic growth and anchorage-independent cell growth assays were performed as previously described (3). For MTS assays, 1 x 10^3^ of HT29, Colo205, or LoVo cells were seeded in 96-well plates, and variant concentrations of drug were added the following day. After a 72 h incubation, cell viability was examined using a CellTiter 96 AQueous Assay kit based on the manufacturer’s instructions (Promega). Briefly, the combined MTS [3-(4,5-dimethylthiazol-2-yl)-5-(3-carboxymethoxyphenyl)-2-(4-sulfophenyl)-2H-tetrazolium, inner salt] and PMS [phenazine methosulfate] solution was added into each well of the cell-containing 96-well plate for 2–3 h at 37 °C. The amount of soluble formazan was measured based on the changes in absorbance at 490 nm using an ELISA plate reader (Multiskan FC, Thermo Fisher Scientific). For clonogenic growth assays, cells were seeded in 6-well plates at a low density. The colonies were stained with crystal violet after 10–14 d. For anchorage-independent cell growth assay, cells were suspended in 0.5% agarose in complete medium and plated on a layer of 0.8% agarose in complete medium in 6-well culture plates. After 3 w, the colonies were stained with 0.02% iodonitrotetrazolium chloride (Sigma-Aldrich). When applicable, a variant concentration of drug was added into the medium and the medium was replaced every other day. The colony was imaged by microscopy and the size was calculated as volume = [length × (width)^2^] / 2.

**Comet assays**

Oxaliplatin- or mock-treated cells were trypsinized, rinsed with ice-cold PBS, collected by centrifugation, and mixed with low-melting point agarose (SeaPlaque™ Agarose, Lonza). 100 μl of the mixture was applied as a smeared layer on cover slide and allowed to solidify at 4 °C. The cells were lysed for 1 h at 4 °C using lysis buffer containing 100 mM EDTA, 2.5 M NaCl, 10 mM Tris–HCl pH 10, 1% Triton X-100, 10 mM β-glycerophosphate, 10 nM calyculin A, 1 mM Na_3_VO_4_ and protease inhibitors. The slides were immersed for 20 min with alkali buffer containing 300 mM NaOH and 1 mM EDTA pH 13.5 and electrophoresis was conducted at 25 V and 300 mA for 20 min, followed by neutralization with 400 mM Tris–HCl pH 7.5. The samples then were stained with SYBR™ Safe (Invitrogen, 1:20000 in neutralizing buffer) for 20 min and imaged. The length of DNA tails was assessed using OpenComet software (v1.3.1, https://cometbio.org/).

**HR and NHEJ assays**

For PCR-based HR assay, the HR activity was analyzed using a Homologous Recombination assay kit (Norgen Biotek) according to the manufacturer’s instruction. In brief, cells were seeded on 24-well plates (5x10^4^ cells per well) and then transfected with plasmid mix (0.5 μg of dl-1 and dl-2) or the control plasmid (0.5 μg per well) using Xfect transfection reagent (Takara Bio), according to the manufacturer’s protocol. Twenty-two hours after transfection, cellular DNA was isolated using the PureLink Genomic DNA Mini Kit (Invitrogen), according to the manufacturer’s protocol. qPCRs were performed using the Fast SYBR Green Master Mix (Applied Biosystems) on a 7500 Fast Real-Time PCR System (Applied Biosystems). Each sample was tested in triplicates, and the level of recombinant DNA produced in the experimental cells was analyzed using the ΔΔCt method and expressed as a relative HR efficiency to the control cells. For qPCR reaction, 50 ng of DNA was used as a template. Primers were supplied by the HR assay kit, including a set of universal primers amplifying all plasmid DNA was used as control for transfection efficiency and another set of primers amplifying plasmid DNA generated by HR was used to measure repair activity. For the BML-277 treatment, BML-277 (10 μM) was added to the cells 12 h before transfection was performed.

The reporter-based HR repair and NHEJ assays were performed in DR-GFP (Direct Repeat-GFP) cells and EJ5-GFP (End Joining-GFP) cells, respectively. DR-GFP cells were generated by transfection of pDR-GFP using Xfect transfection reagent (Takara Bio) and puromycin selection. EJ5-GFP cells were generated by introduction of pimEJ5-GFP (5) and puromycin selection. The fluorescent value of GFP-positive cells was determined by flow cytometry (excitation at 488 nm, FL1: 530/30 filter, FL2: 585/42 nm filter, BD FACSCalibur).

For I-SceI-induced DSBs, 1 x 10^5^ cells LoVo-OR cells carrying DR-GFP or EJ5-GFP were seeded on 12-well plate and transiently transfected with 3 μg I-SceI-expressing plasmid pCAG-I-SceI or an empty vector pCAG using Xfect transfection reagent (Takara Bio). The cells were recovered with fresh medium 4 hr after transfection were performed. The transient transfection efficiency was determined by transfection of 3 μg RFP-expressing plasmid pDsRed in the LoVo-OR stable clones. Seventy-two hours after transfection, the percentages of GFP-positive or RFP-positive cells were determined by flow cytometry. To calculate the efficiency of I-SceI-induced DSB repair, the percentages of GFP-positive cells in each group were normalized with the transfection efficiency value obtained from the cells transfected with pDsRed. For the BML-277 treatment, the transfected cells were recovered with medium containing mock reagent or 10 μM BML-277 until the time of flow cytometric analysis. FlowJo software (BD Biosciences) was used for gate adjusting and plot representation.

**Sister chromatid exchange (SCE)**

SCE analysis was performed as described (6, 7). 1x10^6^ cells were incubated with 9 μg/mL 5-bromodeoxyuridine (BrdU) (Sigma) for 48 h, followed by 0.1 μg/ml colcemid (Thermo Fischer Scientific) treatment for 40 min before standard metaphase chromosome harvest. Images were acquired by Nikon eclipse 80i / NIS Elements D4.20.00. For each cell line, 50 metaphases were analyzed to determine the SCE frequency.

**Immunofluorescence microscopy**

Cells plated on two-well chamber slides were treated with 10 μM oxaliplatin as indicated. Cells were fixed with 3.5% paraformaldehyde for 15 min and permeabilized with 1% Triton X-100 for 10 min. The fixed cells were blocked with 5% FBS and immunolabeled with primary antibodies against γ-H2AX at 4 °C overnight, and then incubated with Alexa Fluor 594-conjugated anti-rabbit IgG (Thermo Fisher Scientific) in the dark. The sections were then counterstained with Hoechst 33342 (Thermo Fisher Scientific) and mounted with fluorescence mounting medium (Dako). Fluorescence microscopy was performed on a NIKON TE2000EPS-C1-S1 microscope. The fluorophores were excited as follows: Hoechst 33342 at 408 nm and Alexa Fluor 594 at 543 nm. Hoechst 33342 emission was collected at 450 ± 35 nm and Alexa Fluor 594 at 590 ± 50 nm. The microscope setting parameters were kept constant and imaged on the same day. Images were taken from a minimum of four stained slides by using 63 × objective lenses. The numbers of γ-H2AX foci in nucleus were counted for 100 cells in each condition using ImageJ (NIH) and plotted using Prism 8 (GraphPad Software).

**Supplementary references**

1. Matsuoka S, Ballif BA, Smogorzewska A, McDonald ER, 3rd, Hurov KE, Luo J, et al. ATM and ATR substrate analysis reveals extensive protein networks responsive to DNA damage. Science. 2007;316(5828):1160-6.

2. Shen CH, Hsieh CC, Jiang KY, Lin CY, Chiang NJ, Li TW, et al. AUY922 induces retinal toxicity through attenuating TRPM1. J Biomed Sci. 2021;28(1):55.

3. Shen CH, Kim SH, Trousil S, Frederick DT, Piris A, Yuan P, et al. Loss of cohesin complex components STAG2 or STAG3 confers resistance to BRAF inhibition in melanoma. Nat Med. 2016;22(9):1056-61.

4. Pierce AJ, Johnson RD, Thompson LH, Jasin M. XRCC3 promotes homology-directed repair of DNA damage in mammalian cells. Genes Dev. 1999;13(20):2633-8.

5. Bennardo N, Cheng A, Huang N, Stark JM. Alternative-NHEJ is a mechanistically distinct pathway of mammalian chromosome break repair. PLoS Genet. 2008;4(6):e1000110.

6. Su WP, Hsu SH, Wu CK, Chang SB, Lin YJ, Yang WB, et al. Chronic treatment with cisplatin induces replication-dependent sister chromatid recombination to confer cisplatin-resistant phenotype in nasopharyngeal carcinoma. Oncotarget. 2014;5(15):6323-37.

7. Su WP, Ho YC, Wu CK, Hsu SH, Shiu JL, Huang JC, et al. Chronic treatment with cisplatin induces chemoresistance through the TIP60-mediated Fanconi anemia and homologous recombination repair pathways. Sci Rep. 2017;7(1):3879.

**Supplementary Figure Legend**

**Supplementary figure 1**

(a) Representative images of clonogenic growth in the oxaliplatin treatment experiments on LoVo (top) and HT29 (bottom) cells. P: parental oxaliplatin-sensitive cells; OR: oxaliplatin-resistant cells. *n* = 3.

(b) Representative images of Human Phospho-Kinase Array. Cell lysates from HT29-P and HT29-OR were prepared and analyzed using Human Phospho-Kinase Array. The protein spots with difference expression levels between oxaliplatin-resistant cells and their parental counter parts were indicated.

(c) Representative western blots of a panel of CRC cell lines (top) and quantification analysis (bottom). Data are mean ± SEM. The *P* values were determined by unpaired two-tailed Student’s t-test, **P* < 0.05, ***P* < 0.01, n.s. = not significant. β-Actin served as a loading control. *n* = 3.

(d) Representative western blots of cells treated with oxaliplatin (top) and quantification analysis (bottom). Data are mean ± SEM. The *P* values were determined by unpaired two-tailed Student’s t-test, **P* < 0.05, ***P* < 0.01, n.s. = not significant. β-Actin served as a loading control. *n* = 3.

**Supplementary figure 2**

(a) Representative images of sister chromatid exchange (SCE) assay in CRC cell lines. SCEs were indicated by arrows.

(b) Representative western blots of the CRC cell lines (left) and quantification analysis (right). Data are mean ± SEM. The *P* values were determined by unpaired two-tailed Student’s t-test, **P* < 0.05, ***P* < 0.01. α-Tubulin served as a loading control. *n* = 3.

(c) A diagram for the end use assay based on the DR-GFP and EJ5-GFP reporter. *SceGFP*: green fluoreScent protein (GFP) gene repeat (SceGFP) which is nonfunctional due to the replacement of 11 bp of GFP sequence to create the18 bp recognition sequence for the I-SceI endonuclease. *iGFP*: internal GFP fragment (iGFP) that can be used to correct the mutation in the *SceGFP* gene to result in a GFP^+^ gene. HR: Homologues recombination. *puroR*: puromycin resistance gene. NHEJ: Non-homologous end joining.

(d) Representative flow cytometry plots. The cells stable expressing pDR-GFP or pimEJ5-GFP were subjected for flow cytometric analysis (excitation at 488 nm, FL1: 530/30 filter, FL2: 585/42 nm filter, BD FACSCalibur). The percentage of GFP-positive cells was shown. *n* = 3.

**Supplementary figure 3**

Representative images of comet assay in oxaliplatin treatment experiments on HT29-P cells stably carrying the empty vector or expressing F-CHK2 constructs as indicated. Scale bar: 50 μm. *n* = 3.

**Supplementary figure 4**

(a) Representative western blots of Olaparib treatment experiments on HT29-OR cells (left) and quantification analysis (right). Data are mean ± SEM. The *P* values were determined by unpaired two-tailed Student’s t-test, **P* < 0.05, ***P* < 0.01, n.s. = not significant. *n* = 3.

(b) Viability of OR CRC cell lines after treatment with various concentrations of Olaparib. Data are mean ± SEM. *n* = 3.

(c) Representative images of tumor spheres. HT29-P cells stably carrying the empty vector or expressing F-CHK2 constructs were grown as tumor sphere in the present of mock reagent or oxaliplatin for 3 wk. Scale bar: 100 μm, *n =* 3.

(d) The growth curves (left) and spider plots (right) of xenograft tumors presented in Figure 5f.

(e) Quantification of body weight of the tumor-bearing mice described in Figure 5f.

**Supplementary figure 5**

(a) Quantification analysis, related to Figure 6a, was performed for three independent experiments. Data are presented as the mean ± SEM. The *P* values were determined by unpaired two-tailed Student’s t-test, **P* < 0.05, **P < 0.01, ***P < 0.001.

(b) Representative images of clonogenic growth assays, anchorage-independent growth assays, and tumor sphere in CRC cells treated with varying concentration of BML-277. LoVo-OR cells were seeded in p60 for clonogenic growth assays and in 6-well plate for anchorage-independent growth assays. Colo205-OR cells were seeded in 6-well plate for clonogenic growth assays. Scale bar: 100 μm. *n* = 3.

(c) The growth curves (left), spider plots (middle), and waterfall plots (right) of xenograft tumors derived from Colo205-P and Colo205-OR cells. The mice bearing Colo205-P tumor were treated with mock (*n* = 8), or administrated 5mg/kg oxaliplatin once per week (*n* = 8). The mice bearing Colo205-OR tumor were treated with 5mg/kg oxaliplatin once per week (*n* = 8). Data are mean ± SEM. The *P* values were determined by unpaired two-tailed Student’s t-test, **P* < 0.05, ***P* < 0.01.

(d) Quantification of body weight of the tumor-bearing mice described in Figure 6c (right) and in supplementary Figure 5c (left).

**Supplementary figure 6**

A schematic representation of the mechanism of CHK2 involves in the development of oxaliplatin resistance that could be inhibited by a CHK2 inhibitor BML-277.
