# Supplementary figures and images for: CHK2 activation contributes to the development of oxaliplatin resistance in colorectal cancer
Source: Br J Cancer. 2022 Aug 23;127(9):1615–28. doi: 10.1038/s41416-022-01946-9 (PMC9596403; doi:10.1038/s41416-022-01946-9)

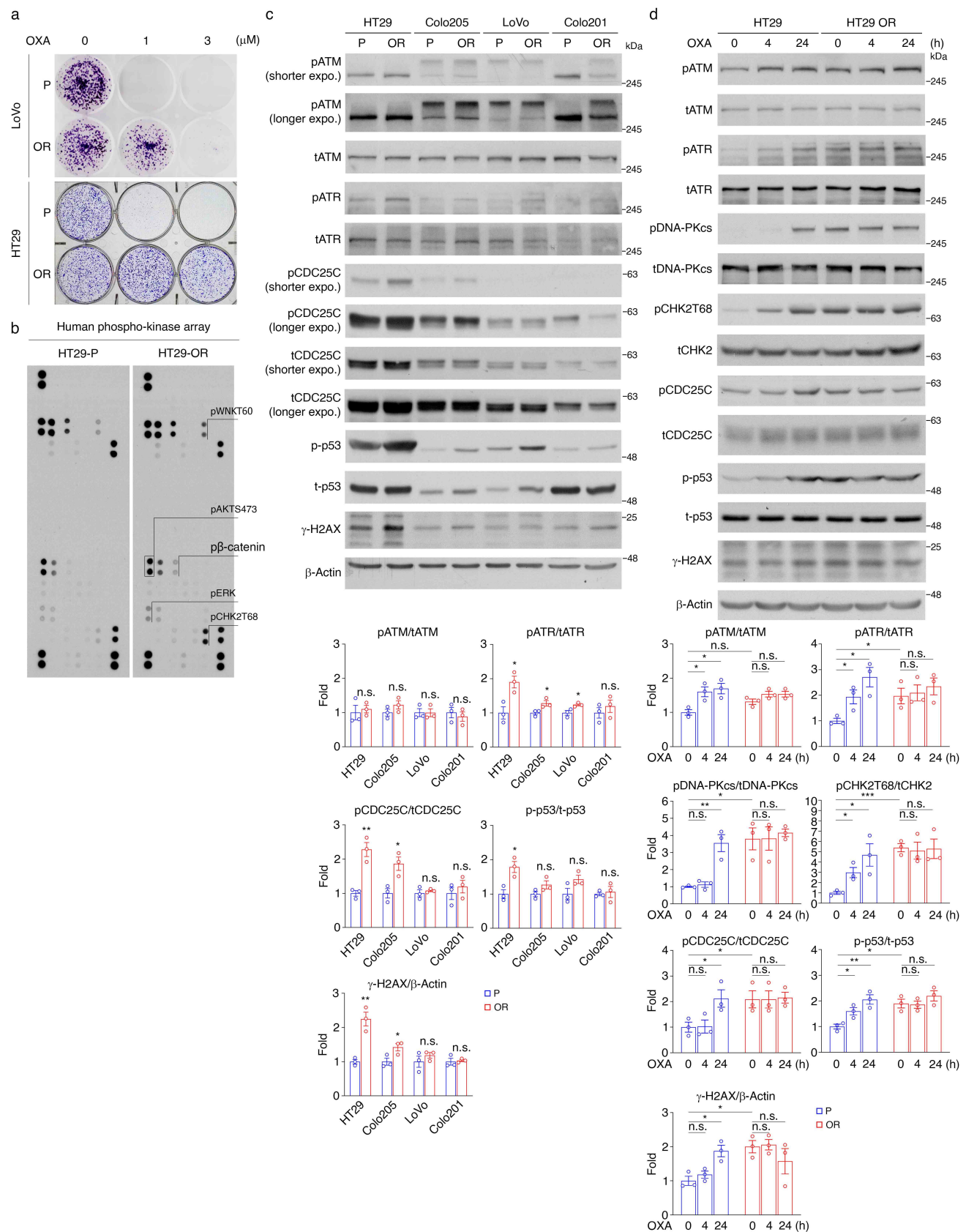

Supplement: Supplementary file 2 — Supplementary figure 1 [file 41416_2022_1946_MOESM2_ESM.pdf]

a

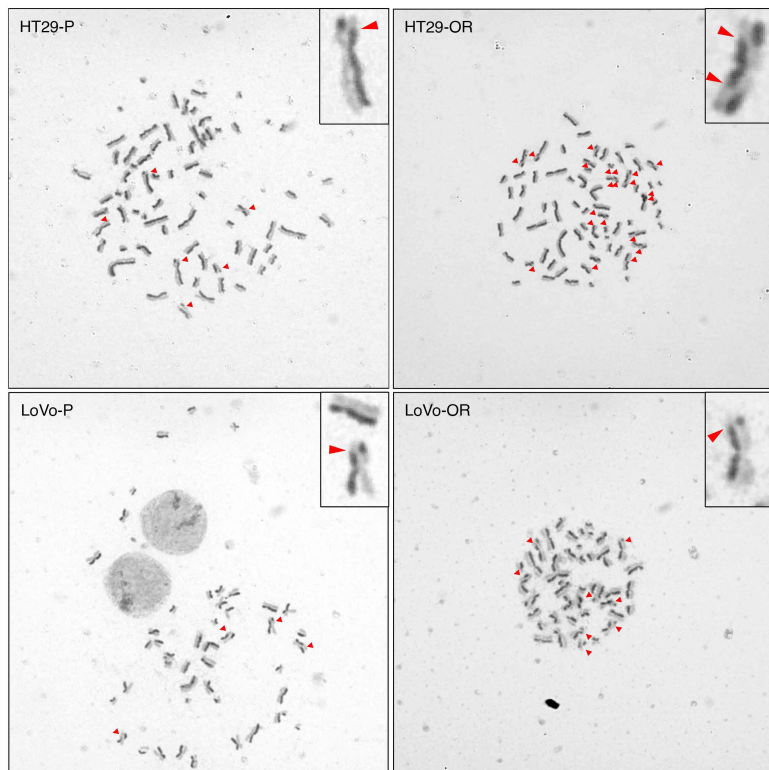

b

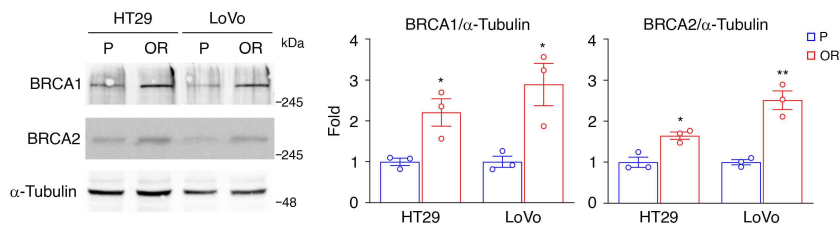

c

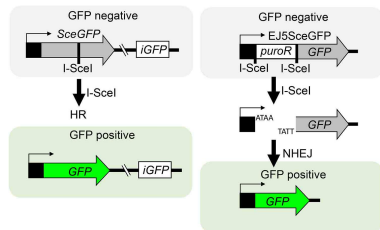

d

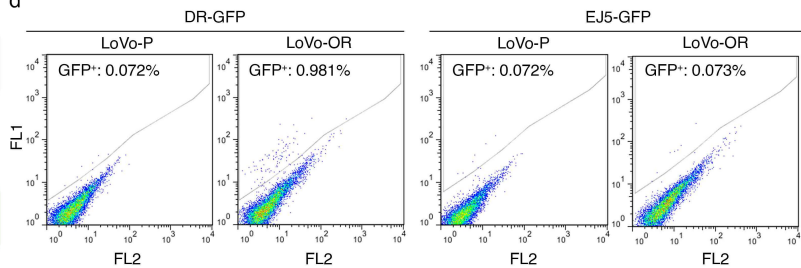

Supplement: Supplementary file 3 — Supplementary figure 2 [file 41416_2022_1946_MOESM3_ESM.pdf]

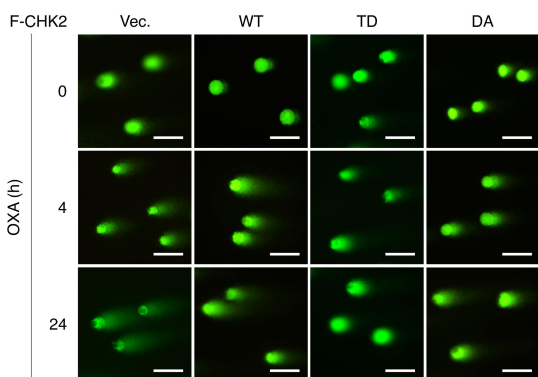

Supplement: Supplementary file 4 — Supplementary figure 3 [file 41416_2022_1946_MOESM4_ESM.pdf]

a

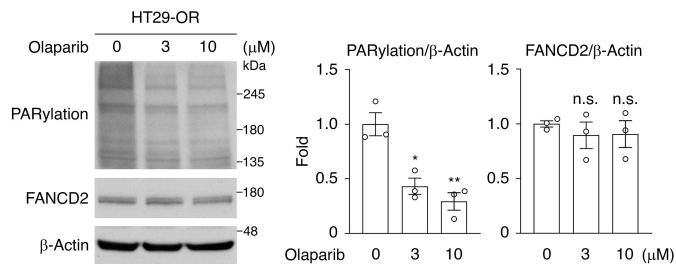

b

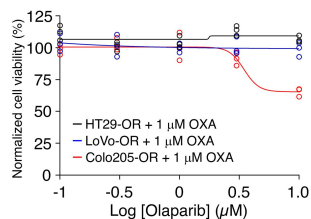

c

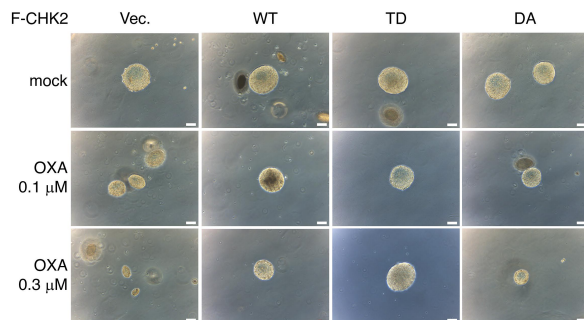

d

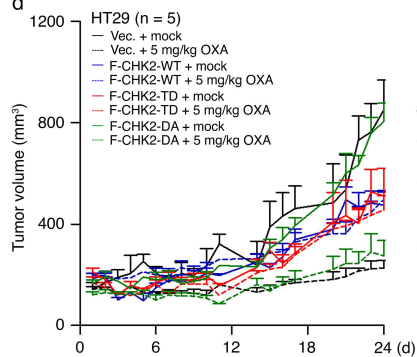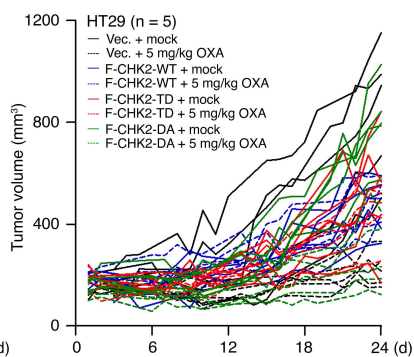

e

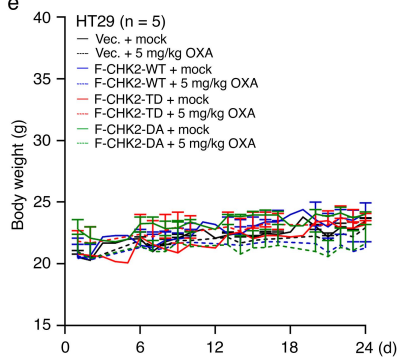

Supplement: Supplementary file 5 — Supplementary figure 4 [file 41416_2022_1946_MOESM5_ESM.pdf]

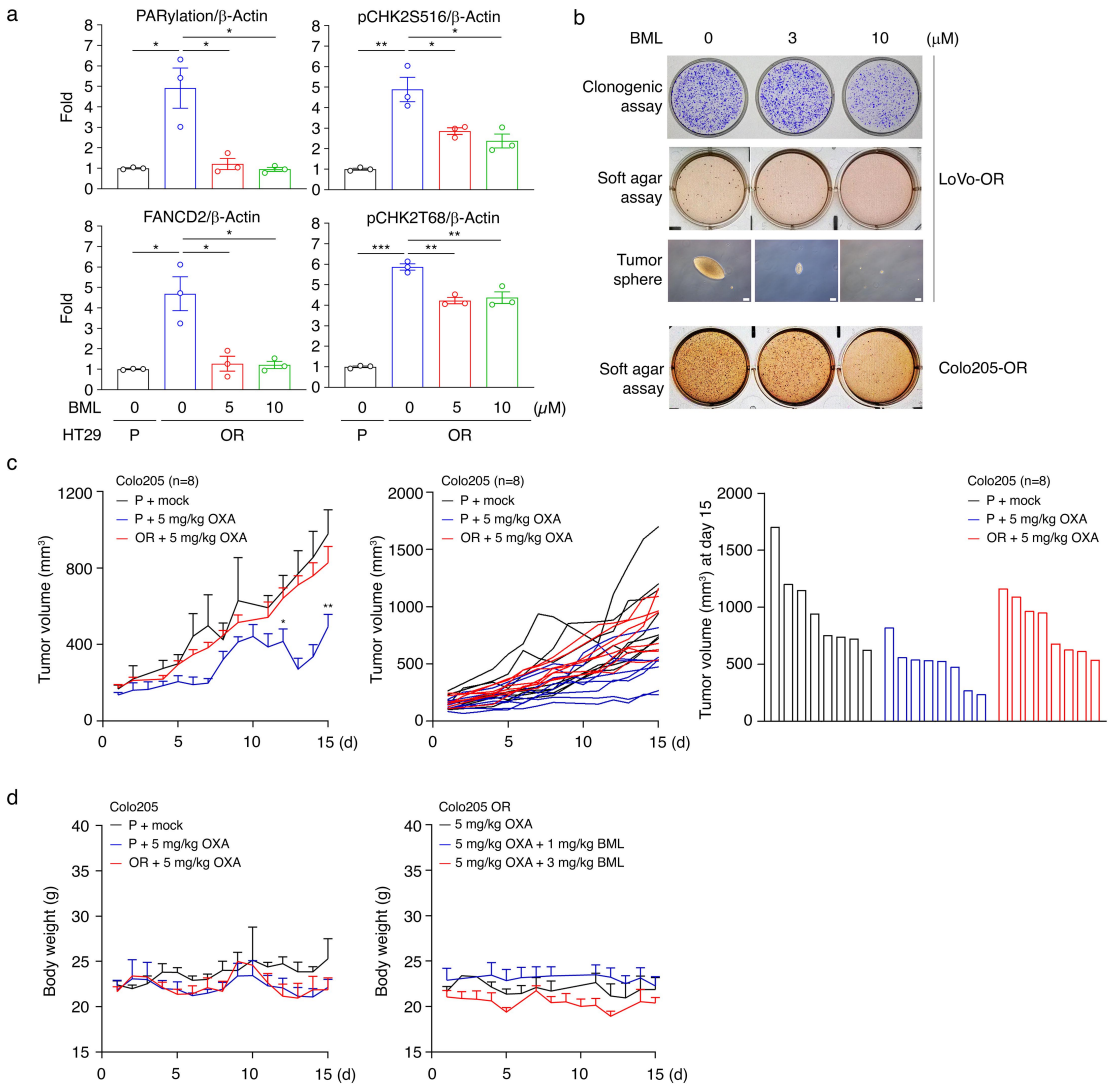

Supplement: Supplementary file 6 — Supplementary figure 5 [file 41416_2022_1946_MOESM6_ESM.pdf]

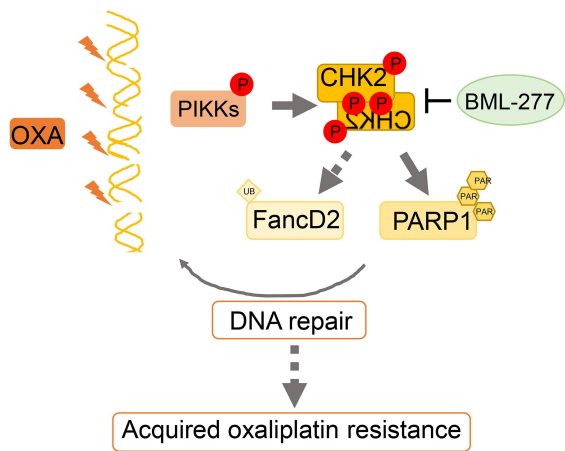

Supplement: Supplementary file 7 — Supplementary figure 6 [file 41416_2022_1946_MOESM7_ESM.pdf]
