# Supplementary material for: CHK2 activation contributes to the development of oxaliplatin resistance in colorectal cancer
Source: Br J Cancer. 2022 Aug 23;127(9):1615–28. doi: 10.1038/s41416-022-01946-9 (PMC9596403; doi:10.1038/s41416-022-01946-9)

Figure 1c

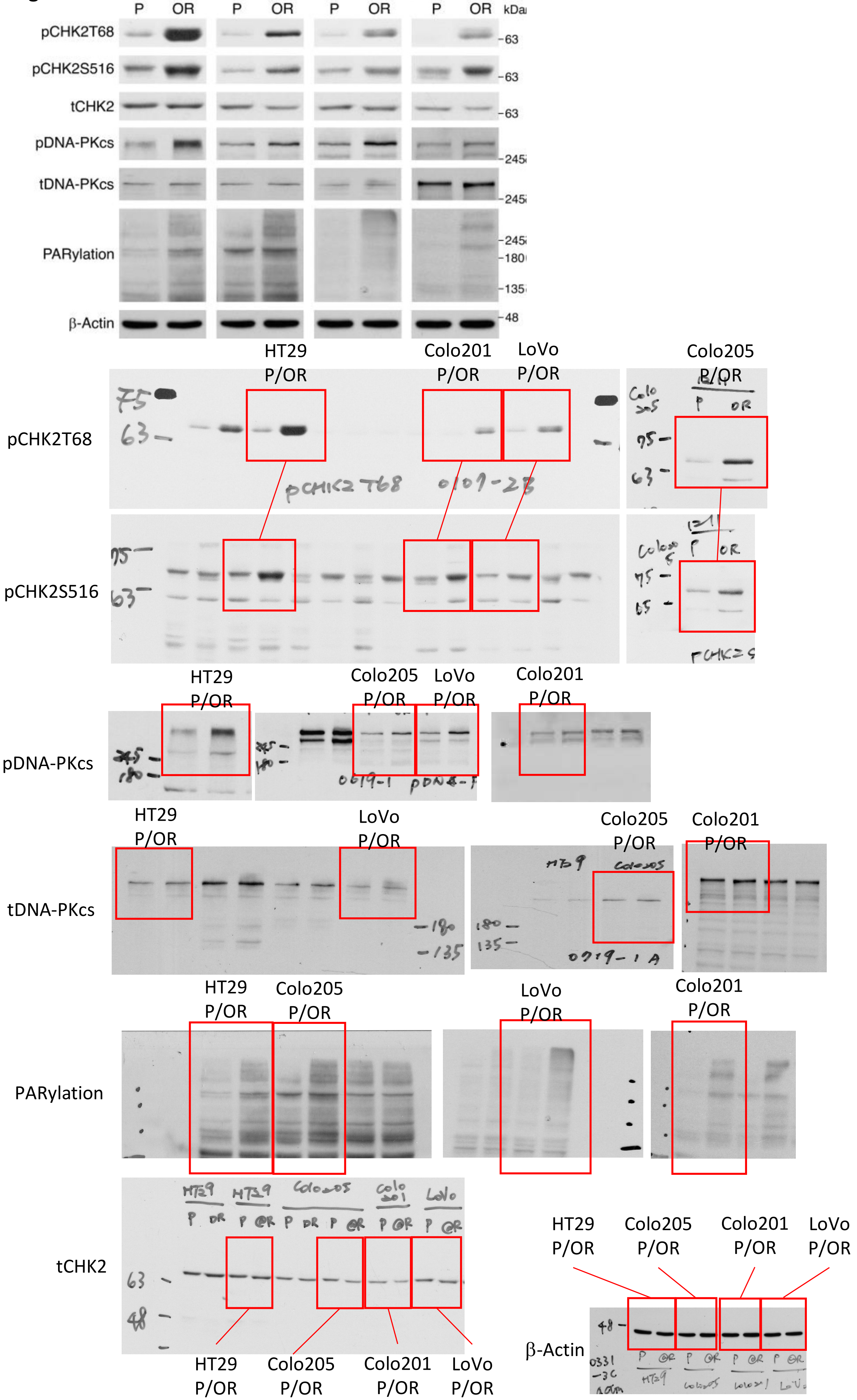

HT29
Colo205
LoVo
Colo201
P
OR
kDa
pCHK2T68
pCHK2S516
tCHK2
pDNA-PKcs
tDNA-PKcs
PARylation
β-Actin
63
245
180
135
48
HT29 P/OR
Colo201 P/OR
LoVo P/OR
Colo205 P/OR
pCHK2T68
pCHK2S516
pDNA-PKcs
tDNA-PKcs
PARylation
tCHK2
β-Actin

Figure 3a

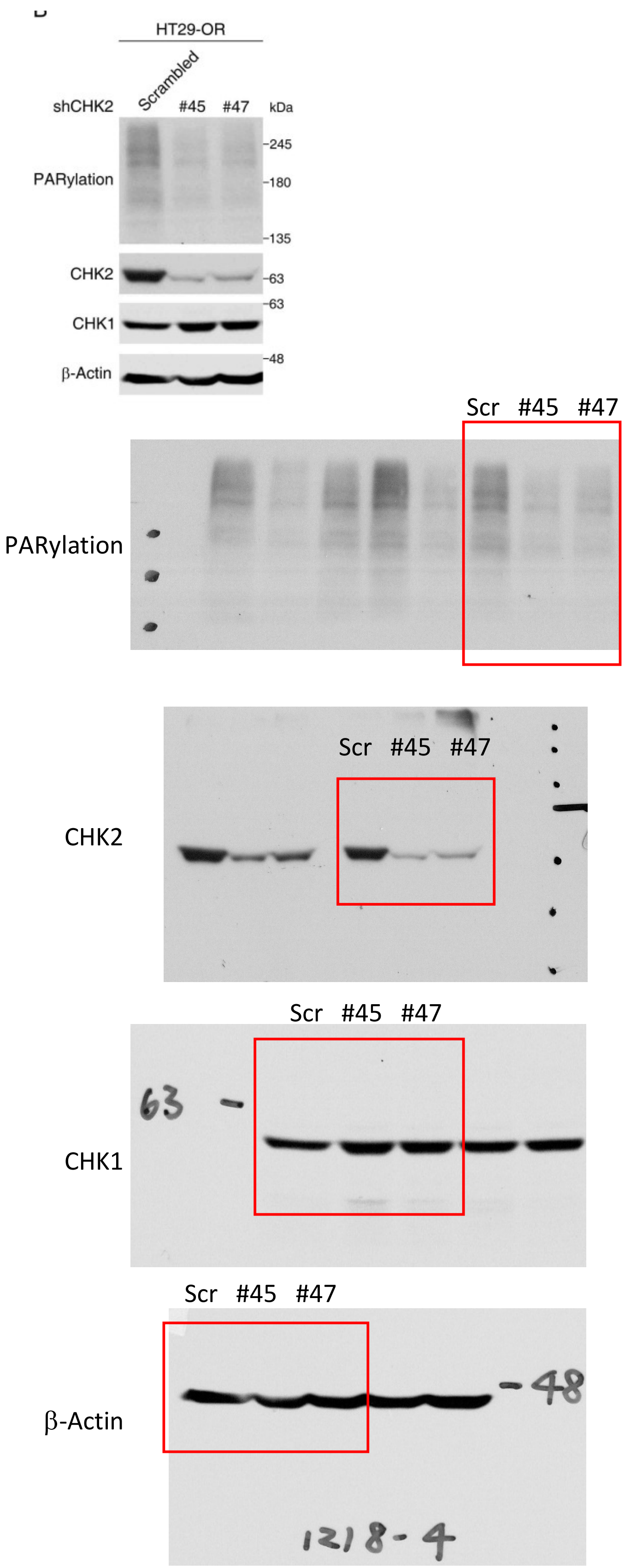

Figure 3b

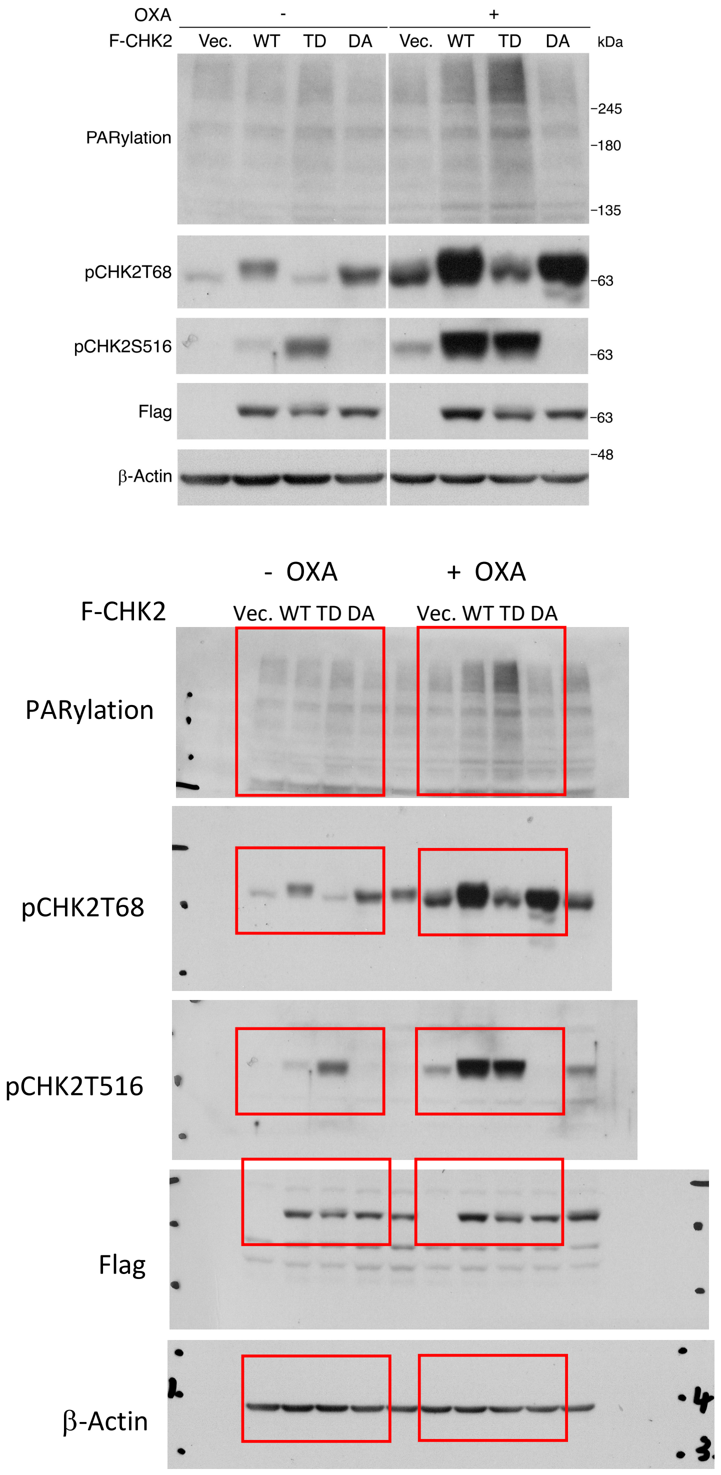

Figure 3d

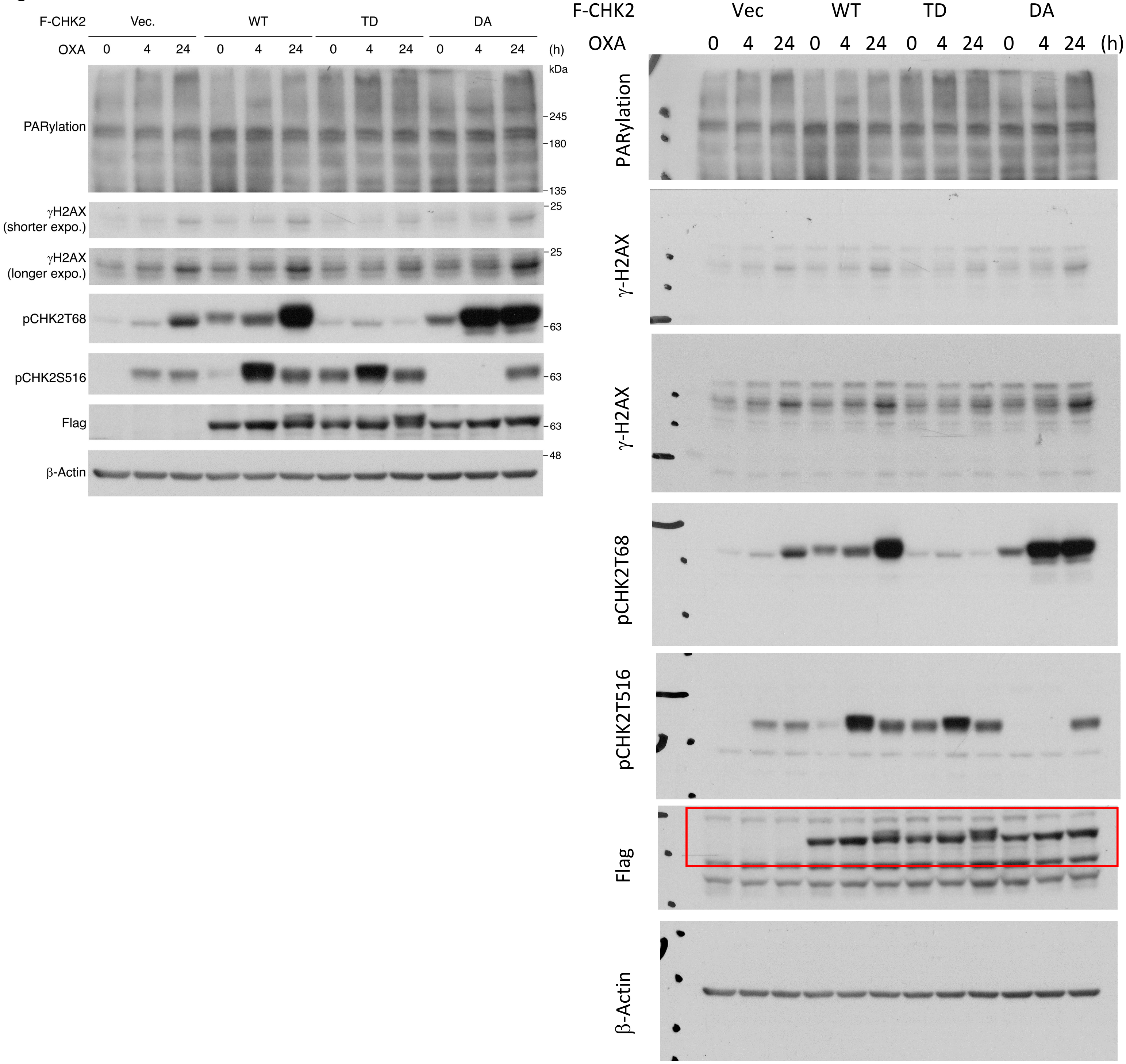

F-CHK2
Vec.
WT
TD
DA
OXA
0
4
24
(h)
kDa
PARylation
245
180
135
γH2AX (shorter expo.)
25
γH2AX (longer expo.)
pCHK2T68
63
pCHK2S516
Flag
β-Actin
48
Vec
OXA
γ-H2AX
pCHK2T516

Figure 4a

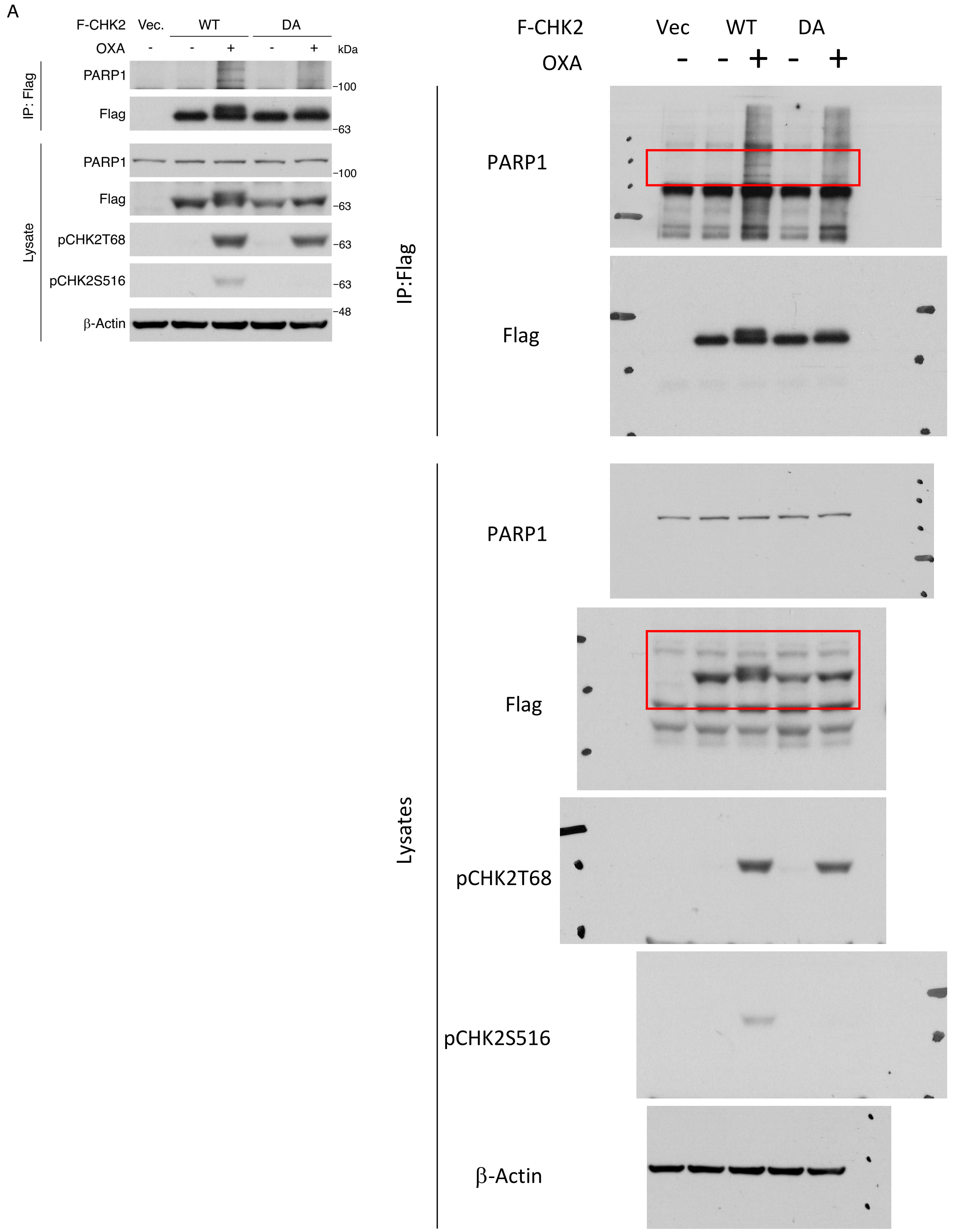

A
F-CHK2
Vec.
WT
DA
OXA
kDa
IP: Flag
PARP1
Flag
Lysate
PARP1
Flag
pCHK2T68
pCHK2S516
β-Actin
100
63
48
F-CHK2
Vec
WT
DA
OXA
IP:Flag
PARP1
Flag
Lysates
PARP1
Flag
pCHK2T68
pCHK2S516
β-Actin

Figure 4b

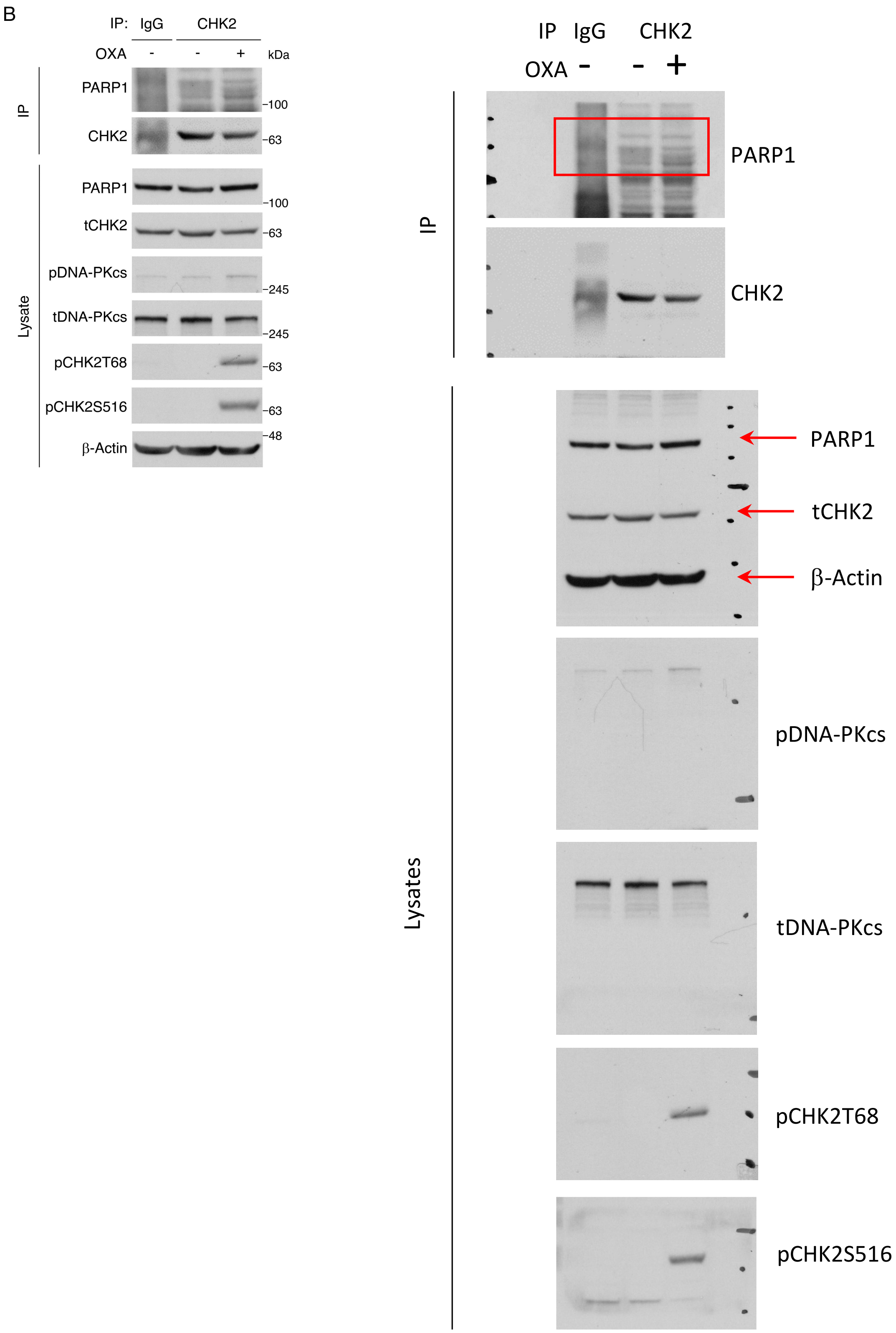

B
IP: IgG CHK2
OXA - - +
kDa
IP
PARP1
CHK2
Lysate
PARP1
tCHK2
pDNA-PKcs
tDNA-PKcs
pCHK2T68
pCHK2S516
β-Actin
100
63
100
63
245
245
63
63
48
IP IgG CHK2
OXA - - +
IP
PARP1
CHK2
Lysates
PARP1
tCHK2
β-Actin
pDNA-PKcs
tDNA-PKcs
pCHK2T68
pCHK2S516

Figure 4c

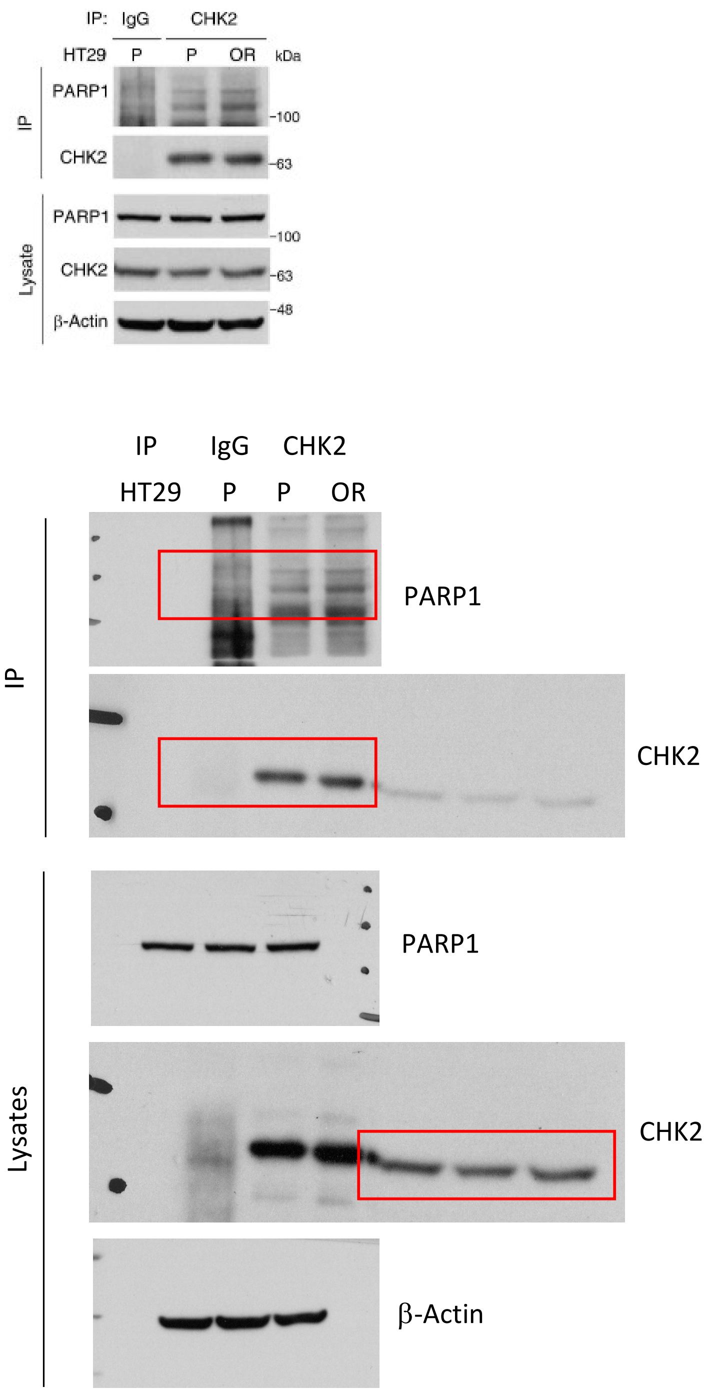

Figure 4d

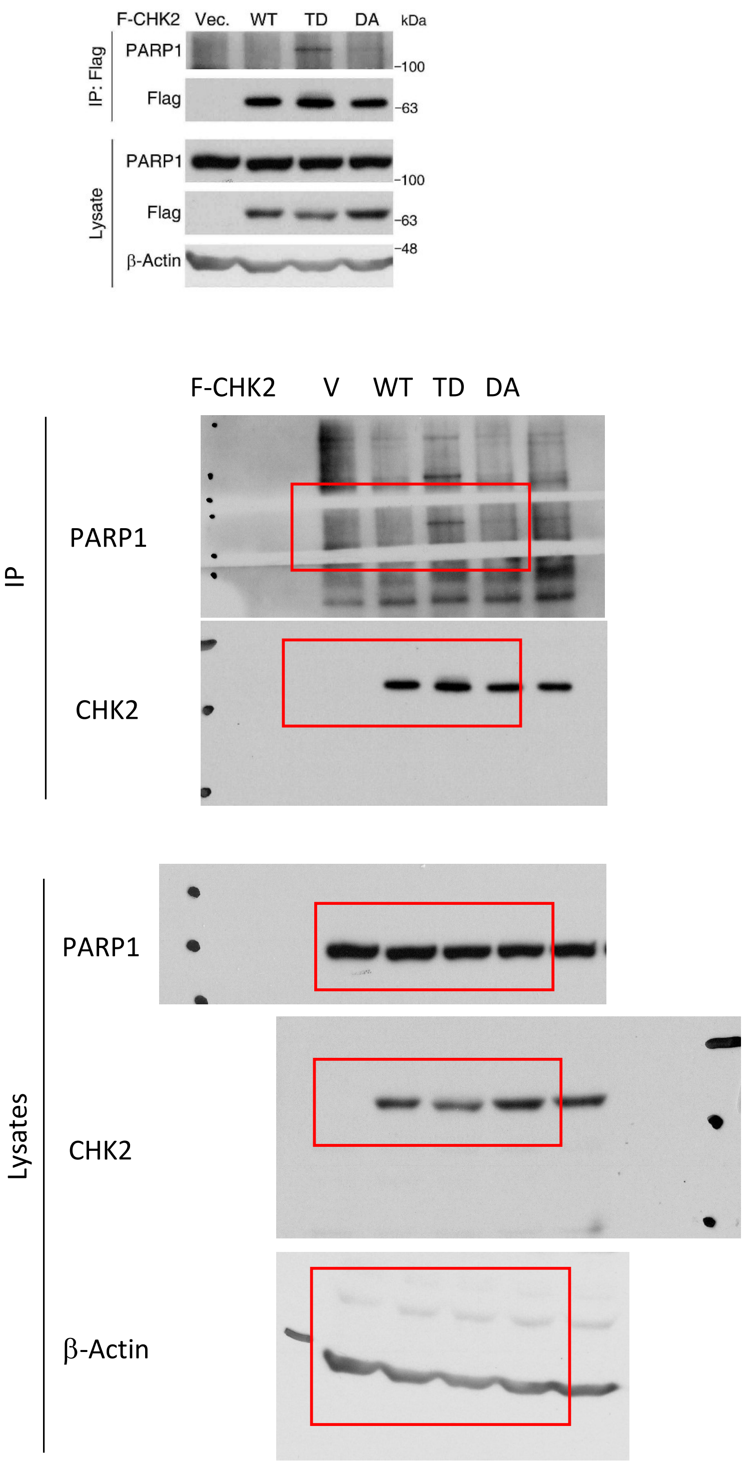

Figure 4e

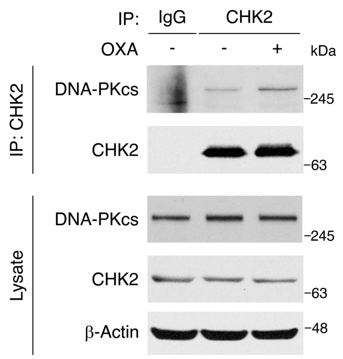
IP: IgG CHK2
OXA - - +
kDa
IP: CHK2
DNA-PKcs
-245
CHK2
-63
Lysate
DNA-PKcs
-245
CHK2
-63
β-Actin
-48

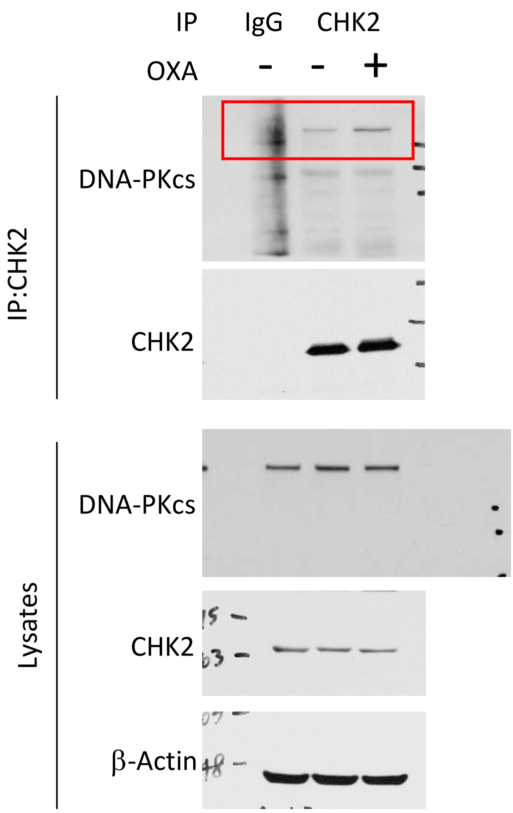
IP IgG CHK2
OXA - - +
IP:CHK2
DNA-PKcs
CHK2
Lysates
DNA-PKcs
CHK2
β-Actin

Figure 5a

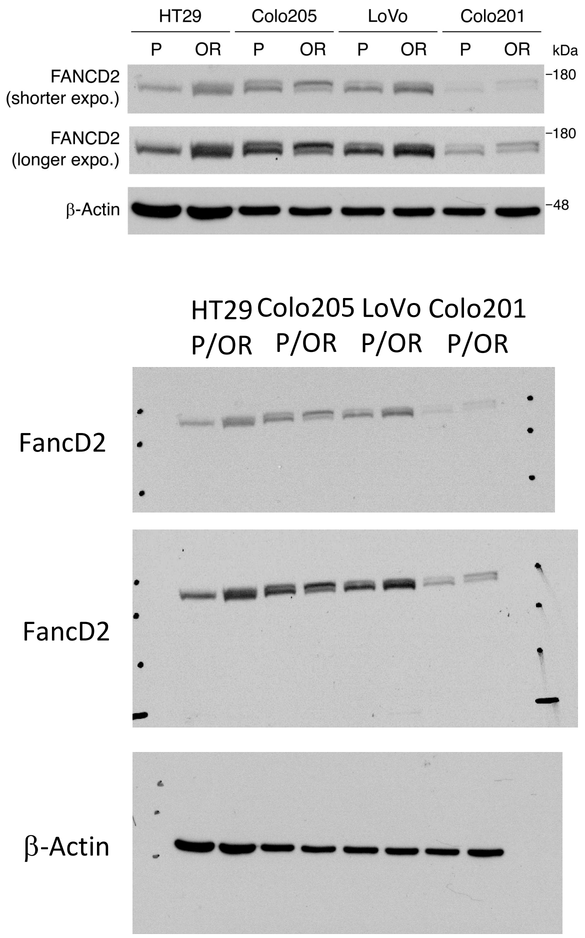

Figure 5b

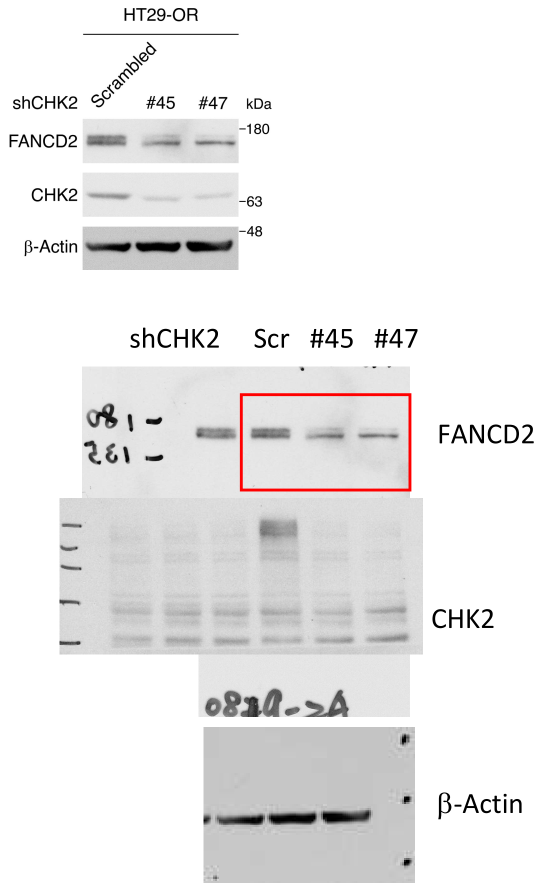

Figure 6a

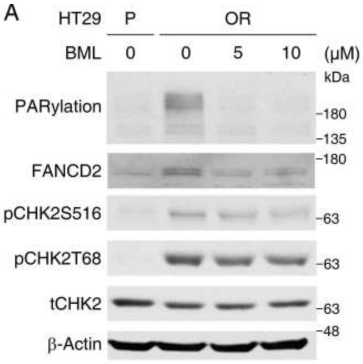
A
HT29 P OR
BML 0 0 5 10 (μM)
kDa
PARylation
–180
–135
FANCD2
–180
pCHK2S516
–63
pCHK2T68
–63
tCHK2
–63
β-Actin
–48

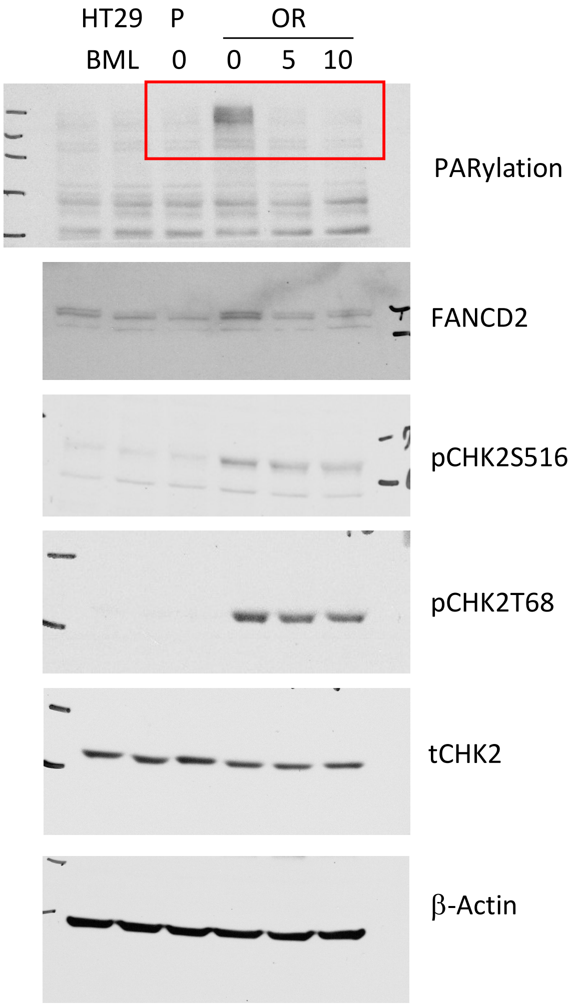
HT29 P OR
BML 0 0 5 10
PARylation
FANCD2
pCHK2S516
pCHK2T68
tCHK2
β-Actin

Supplementary figure 1c

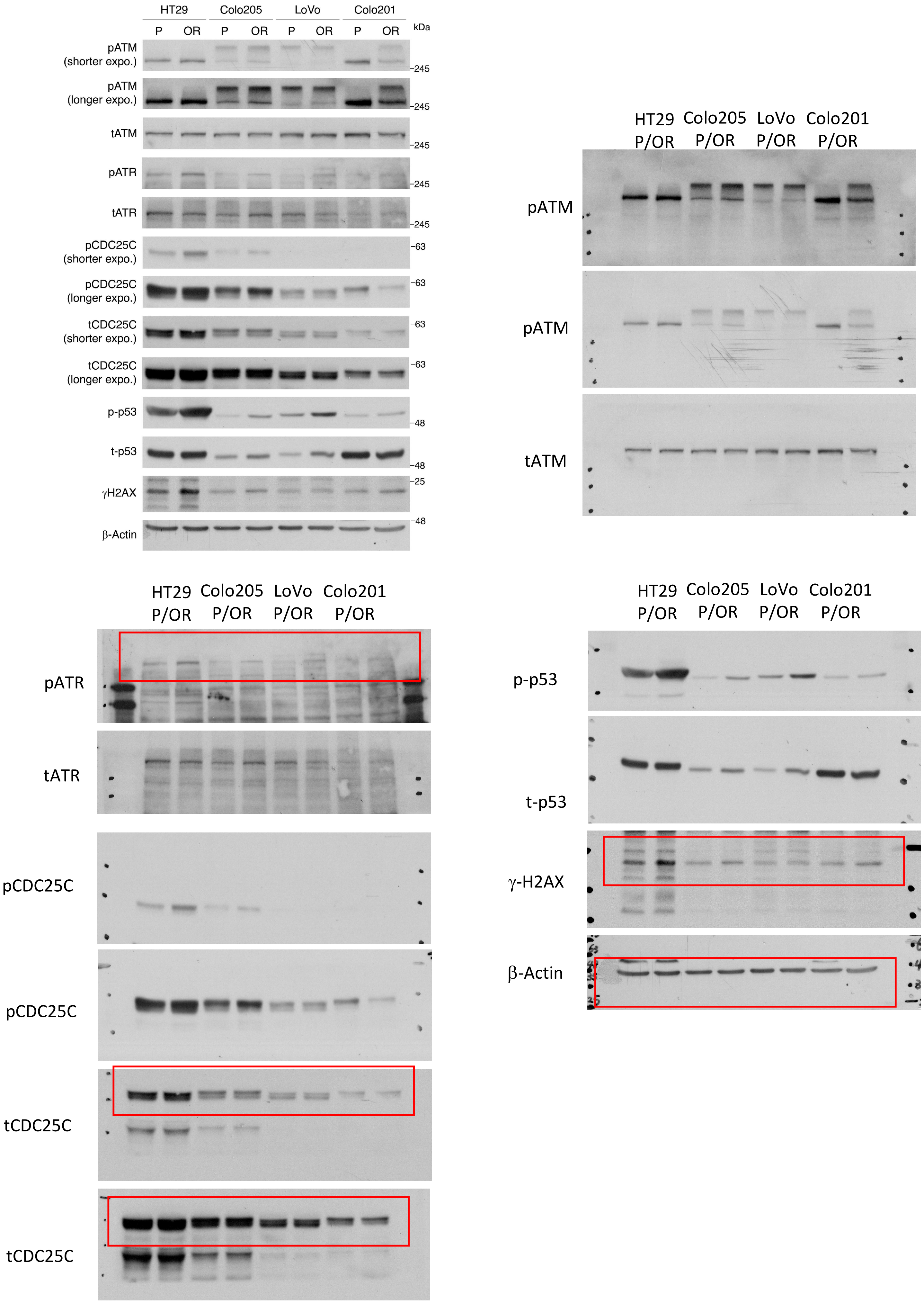

Supplementary figure 1d

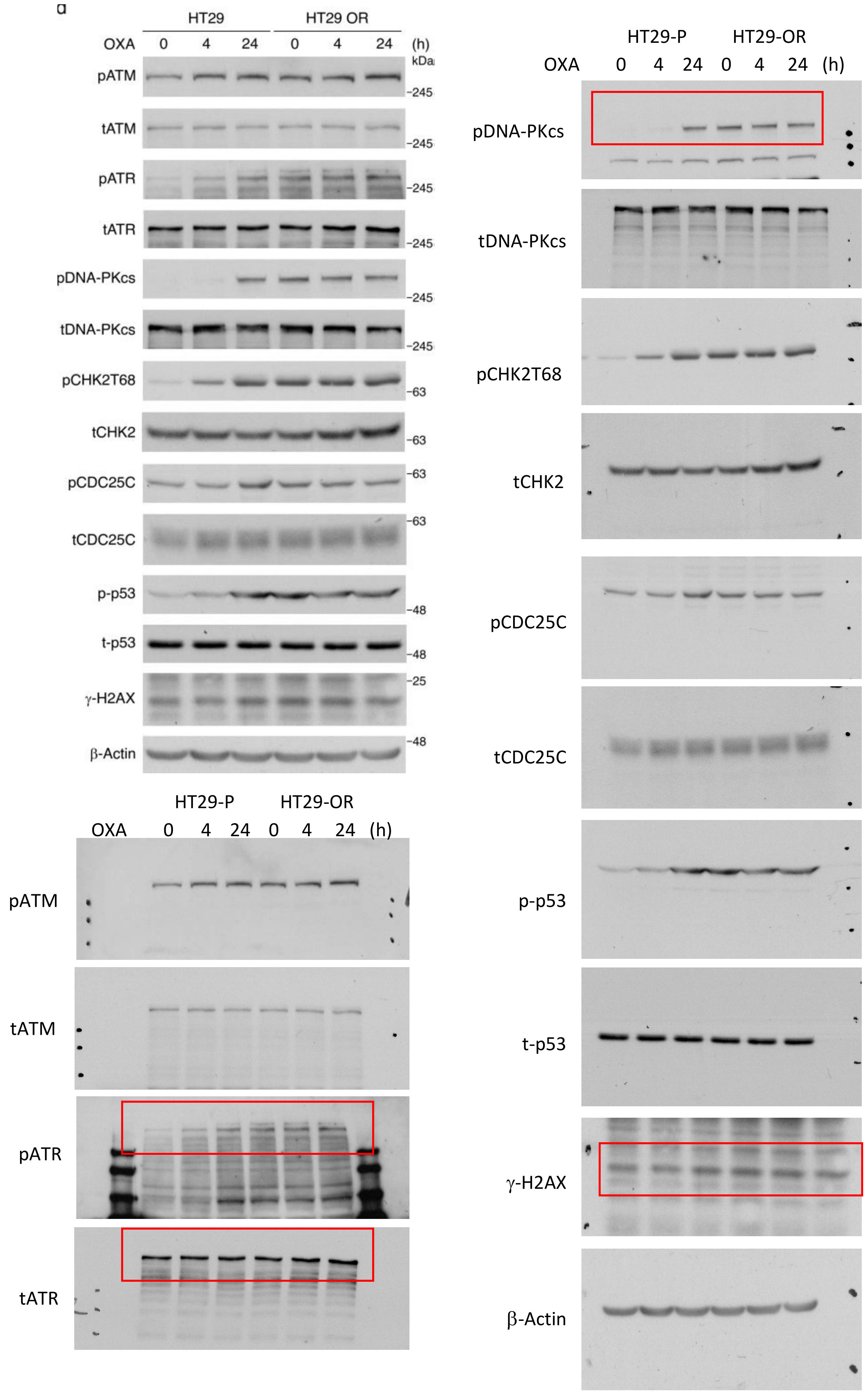

Supplementary figure 2b

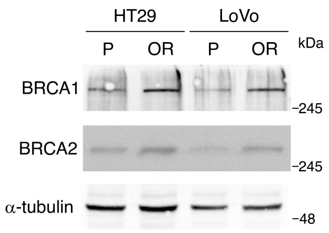

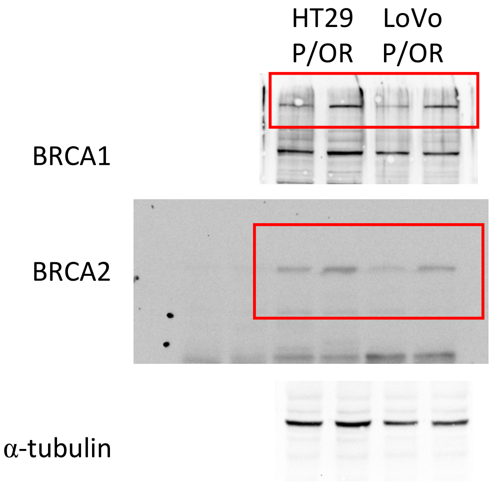

Supplementary figure 4a

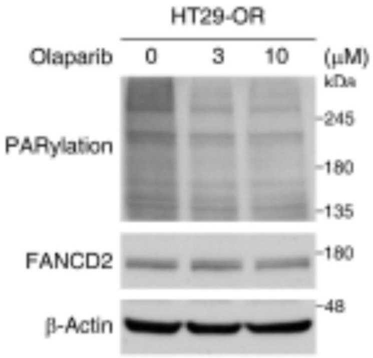

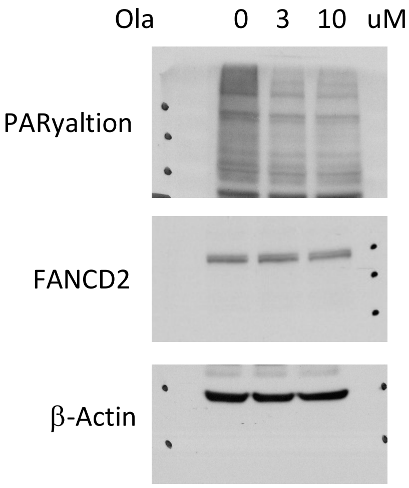

Supplement: Supplementary file 8 — Non-Cropped WB Images [file 41416_2022_1946_MOESM8_ESM.pdf]
